# DEPTOR stabilizes ErbB2 to promote the proliferation and survival of ErbB2-positive breast cancer cells

**DOI:** 10.7150/thno.51286

**Published:** 2021-04-19

**Authors:** Yanli Bi, Xiaoyu Chen, Bajin Wei, Linchen Wang, Longyuan Gong, Haomin Li, Xiufang Xiong, Yongchao Zhao

**Affiliations:** 1 Department of Hepatobiliary and Pancreatic Surgery, the First Affiliated Hospital, Zhejiang University School of Medicine, Hangzhou, China.; 2 Zhejiang Provincial Key Laboratory of Pancreatic Disease, the First Affiliated Hospital, Zhejiang University School of Medicine, Hangzhou, China.; 3 Institute of Translational Medicine, Zhejiang University School of Medicine, Hangzhou, China.; 4 Cancer Institute of the Second Affiliated Hospital, Zhejiang University School of Medicine, Hangzhou, China.; 5 Department of Breast Surgery, the First Affiliated Hospital, Zhejiang University School of Medicine, Hangzhou, China.; 6 Children's Hospital, Zhejiang University School of Medicine, Hangzhou, Zhejiang, China.; 7 Cancer Center, Zhejiang University, Hangzhou, China.; 8 Department of Medical Oncology, the First Affiliated Hospital, Zhejiang University School of Medicine, Hangzhou, China.

**Keywords:** ErbB2, DEPTOR, β-TrCP, PDZ, breast tumorigenesis

## Abstract

**Rationale:** Dysregulation of the PI3K/AKT/mTOR pathway occurs frequently in cancers, providing an attractive therapeutic target for anticancer treatments. DEPTOR plays essential roles in regulation of cell proliferation and survival by directly modulating mTOR activity. However, whether DEPTOR regulates the growth of ErbB2-positive breast cancer cells remains unknown.

**Methods:** DEPTOR expression was determined by TCGA data analysis and immunohistochemistry of human breast tissue microarrays. The membrane localization of DEPTOR was demonstrated by immunofluorescence and subcellular fractionation. The interaction of DEPTOR with ErbB2 was determined by immunoprecipitation. Furthermore, the biological significance of this interaction was assessed by ATPlite cell growth, clonogenic survival, and flow cytometry-based apoptosis assays.

**Results:** DEPTOR promoted the proliferation and survival of ErbB2-positive breast cancer cells by directly interacting with and stabilizing ErbB2. Specifically, DEPTOR translocates to cell membrane and interacts with ErbB2 to disrupt ErbB2 polyubiquitination and degradation promoted by β-TrCP, an E3 ubiquitin ligase. DEPTOR knockdown destabilizes ErbB2 by shortening its protein half-life to inactivate ErbB2-PI3K-AKT-mTOR signaling, leading to the suppression of cell proliferation and survival by inducing apoptosis. Ectopic expression of a constitutively active ErbB2 mutant completely rescued the reduction in cell proliferation and survival by DEPTOR knockdown. Importantly, DEPTOR expression is increased in human breast cancer tissues and its overexpression correlates with poor patient survival. Moreover, DEPTOR is located on the cell membrane in ErbB2-positive breast cancer tissues, but not in tumor-adjacent normal tissues, indicating that DEPTOR may contribute to the oncogenic characteristics of ErbB2.

**Conclusions:** Our study reveals a novel mechanism by which DEPTOR promotes breast cancer cell proliferation and survival by stabilizing ErbB2.

## Introduction

DEP domain-containing mTOR interacting protein (DEPTOR) is a naturally occurring inhibitor of mTOR that plays essential roles in many cellular processes, including cell proliferation, survival, metabolism, apoptosis, and immunity 1. DEPTOR is composed of two N-terminal tandem DEP (Disheveled, EGL-10, and Pleckstrin) domains and a C-terminal PDZ (PSD95, Dlg, and ZO-1) domain. DEP domains are present in a variety of proteins and mainly function in the control of signal transmission by recruiting proteins to the cell membrane and binding to cell surface receptors or phospholipids in the membrane 2. PDZ domains are often found in multi-domain scaffolding proteins and are involved in both protein-protein interactions and anchoring membrane receptors to cytoskeletal components, thereby playing a vital role in the formation and function of signal transduction complexes 3, 4. The PDZ domain of DEPTOR mediates its interaction with mTOR and suppresses the activity of both mTORC1 and mTORC2 5. Given that mTOR functions as a central regulator of cell proliferation and survival, DEPTOR has the potential to play crucial roles in tumorigenesis 1, 6. Interestingly, DEPTOR can act either as a tumor suppressor or oncogene in a context-dependent manner 1, 6, 7; however, the molecular mechanisms remain elusive.

ErbB2 (epidermal growth factor receptor 2), also known as HER2 and Neu, is a member of the epidermal growth factor receptor family, which includes ERBB1-4. Each ERBB protein consists of an extracellular domain, a single transmembrane helix, and an intercellular kinase domain 8. No ligands have yet been identified for ErbB2, but it can form a heterodimer with either ErbB1 (also known as EGFR) or ErbB3 in response to extracellular stimuli. ErbB2 also forms homodimers when overexpressed, which occurs in 20-30% of breast cancers, leading to ErbB2 activation *via* phosphorylation of its kinase domain 8, 9. In breast cancer, high levels of ErbB2 are correlated with poor prognosis, lymph node metastasis, and drug resistance 9. Thus, elucidating the mechanism underlying the regulation of ErbB2 expression may provide novel insights into the development of ErbB2-targeted anticancer therapies.

In this study, we found that in ErbB2-positive breast cancer tissues and cells, DEPTOR is present on the cell membrane where it interacts with ErbB2 (residues 1026 to 1240) *via* its PDZ domain. This interaction prevents the binding of ErbB2 to its E3 ubiquitin ligase β-TrCP, leading to its stabilization. Depletion of DEPTOR dramatically induces proteasomal degradation of ErbB2 and downregulates the PI3K/AKT/mTOR signaling cascade, inducing apoptosis, and thus reducing cell proliferation and survival, which are completely reversed by the ectopic expression of a constitutively active mutant ErbB2. Thus, our study revealed a previously unknown mechanism related to the positive regulation of breast cancer cell proliferation and survival involving the stabilization of ErbB2 by DEPTOR, suggesting that targeting DEPTOR *via* a variety of means might have therapeutic potential for the treatment of ErbB2-positive breast cancer.

## Materials and Methods

### Cell culture and chemicals

All cell lines used in this study were obtained from American Type Culture Collection (ATCC) and were authenticated by ATCC. Cells were expanded and preserved in liquid nitrogen upon receipt. Cells for experiments were passaged for fewer than 25-30 times. MDA-MB-361, MDA-MB-231, MDA-MB-157, MDA-MB-435, MDA-MB-468, T47D, SK-BR3, MCF7, BT20, HEK293, 293T and BOSC cells were maintained in Dulbecco's modified Eagle's medium (DMEM), supplemented with 10% (v/v) fetal bovine serum (FBS) and 1% (v/v) penicillin-streptomycin at 37 °C in a humidified incubator with 5% CO_2_. BT474, AU565, ZR75-30 and ZR75-1 were maintained in RPMI 1640 medium with 10% FBS and 1% penicillin-streptomycin. MG132 was purchased from Cayman. Chloroquine (CQ) and cycloheximide (CHX) were purchased from Sigma-Aldrich. Rapamycin (HY-10219) was purchased from MedChem Express.

### Immunoblotting and immunoprecipitation

Cells were harvested, lysed and subjected to direct immunoblotting (IB) or immunoprecipitation (IP) as previously described 10. Briefly, cells were lysed in lysis buffer (50 mM Tris pH 7.5, 0.15 M NaCl, 1% NP-40, 0.1% SDS, 0.5% sodium deoxycholate, 50 mM NaF, 1 mM EDTA, 1 mM DTT, 1 mM Na_3_VO_4_) with protease inhibitors (11873580001, Roche) and phosphatase inhibitors (04906837001, Roche), and incubated on ice for 30 min. The supernatants were harvested by spinning at 14,000 rpm for 25 min at 4 ºC. The same amounts of whole cell lysates were subjected to IB after the protein concentration measured using the BCA protein assay kit (23225, Thermo). To immunoprecipitate exogenously expressed FLAG-tagged proteins or endogenous proteins, the supernatants were incubated with anti-FLAG^®^ M2 affinity gel (A2220; Sigma) or according antibodies followed by protein-G beads (17061801, GE healthcare) in a rotating incubator for 3 hrs at 4 °C. The immunoprecipitates were washed with lysis buffer for four times and then subjected to IB. Primary antibodies were used as follows: ErbB2 (2165#), DEPTOR (11816#), β-TrCP (11984#), CHIP (2080#), p-AKT (S473) (4060#), p-AKT (T308) (2965#), t-AKT (4691#), p-S6K1 (T389) (9234#), p-ERK (T202/Y204) (9101#), t-ERK (9107#), mTOR (2972#), Caspase 3 (9662#), PARP (9532#), BIM (2933) (Cell Signaling Technology), c-Cbl (sc-1651), ErbB2 (sc-33684), t-S6K (sc-230) and GFP (sc-9996) (Santa Cruz), ATP1A1 (14418-1-AP) (Proteintech), FLAG (F1804), Tubulin (T9026) and ACTIN (A5441) (Sigma), HA (11867423001) (Roche).

### siRNA and lentivirus-based shRNA silencing

Cells were transfected with the following siRNA oligos by Lipofectamine 2000 (11668019, Invitrogen), according to the manufacturer's instructions. The sequences of siRNA oligonucleotides are as follows: siDEPTOR-1: 5'-GCC ATG ACA ATC GGA AAT CTA-3'; siDEPTOR-2: 5'- CCT ACA TGA TAG AAC TGC CTT-3'; siβ-TrCP1+2: 5′-AAG TGG AAT TTG TGG AAC ATC-3′, and siCtrl: 5'-ATT GTA TGC GAT CGC AGA C-3'. For lentivirus-based shRNA silencing, short hairpins were cloned into pLKO.1-puro vector. Lentiviral shRNA virus packaging and infection were implemented according to the protocol described previously 11. Briefly, 293T cells were transfected with pLKO.1 shRNA vectors, psPAX2, and pMD2.G plasmids to produce lentiviruses. Supernatants with viruses were collected and filtered on a 0.45 μm filter, and then pelleted by centrifugation at 13,000 rpm for 4 hrs at 4 °C. The virus particles were finally resuspended in DMEM.

### Plasmids and retrovirus-based gene ectopic expression

Full-length DEPTOR, DEP domains, and PDZ domain were subcloned into pIRES2 and pEGFP vectors, respectively. Full-length ErbB2, ErbB2 (AA 1-1025), 1-1240 amino acids of ErbB2 and DEPTOR-ΔPDZ were subcloned into pIRES2 vectors. DEPTOR and β-TrCP2 were subcloned into pcDNA3.1-3HA vectors. MDA-MB-231 and SK-BR3, as well as HEK293 cells were transfected with the indicated plasmids using Lipofectamine 3000 (L3000015, Thermo Fisher Scientific) and PolyJet (SL100688, SignaGen Laboratories), respectively, according to the manufacturer's instructions. For retrovirus-based ErbB2 and ErbB2-YVMA expression, pBabe-ErbB2-YVMA purchased from Addgene (40982). Retrovirus packaging and infection were implemented according to the protocol described previously 12. Briefly, BOSC cells were transfected with pBabe-ErbB2-YVMA, GAG, and VSV-G plasmids to produce retroviruses. Supernatants containing viruses were collected and filtered on a 0.45 μm filter, and then pelleted by centrifugation at 13,000 rpm for 4 hrs at 4 °C. Virus particles were finally resuspended in DMEM.

### Subcellular fractionation

Plasma membrane and cytoplasmic fractions were extracted using Minute (TM) Plasma Membrane Protein Isolation and Cell Fractionation Kit (SM-005, Invent), according to the manufacturer's instructions. Briefly, cells were collected and lysed in 1 mM PMSF-containing buffer A for 10 min, followed by vortex for 30 sec at ultrahigh speed. Immediately, cell suspension was transferred to the filter cartridge and centrifuged at 16,000 g for 30 sec for twice at 4 °C. The pellets were resuspended by vigorously vortexing for 10 sec, followed by centrifugation at 3000 rpm for 1 min at 4 °C (intact nuclei). The supernatants were transferred to a fresh 1.5 mL microcentrifuge tube and centrifuged at 16,000 g for 30 min at 4 °C. Remove the supernatant (the cytosol fraction) and save the pellet (the total membrane protein fraction including organelles and plasma membranes). The total membrane protein fraction was resuspended in 200 μL buffer B by repeatedly pipetting up and down or vortexing, followed by centrifugation at 16,000 g for 5 min at 4 °C. The supernatant was carefully transferred to a fresh 2.0 mL microcentrifuge tube and added 1.6 mL cold PBS, followed by centrifugation at 16,000 g for 30 min. The pellets were saved as plasma membrane protein fraction and then dissolved in Non-Denatured Protein Solubilization Reagent (WA-010, Invent). Proteins were separated on SDS-PAGE and immunoblotted.

Crude membrane and cytoplasmic fractions were extracted using Membrane and Cytosol Protein Extraction Kit (P0033, Beyotime), according to the manufacturer's instructions. Briefly, cells were collected and lysed in 1 mM PMSF-containing buffer A for 15 min and freeze-thawed three times with liquid nitrogen. The supernatant was collected to remove the nuclei and unbroken cells by spinning at 700 g for 10 min at 4 °C. The supernatant was then collected as cytoplasm after spinning at 14,000 g for 30 min at 4 °C. The cell membrane fractions were re-suspended in buffer B and incubated on ice for 15 min, followed by vortex for 5 sec at ultrahigh speed for three times. Then membrane proteins-containing supernatants were collected by centrifugation at 14,000 g for 5 min at 4 °C. Proteins were separated on SDS/PAGE and immunoblotted.

### ATPlite-based cell growth and clonogenic survival assays

Cells were seeded in 96-well plates in triplicate at 3000 cells per well. Cell proliferation was determined by an ATPlite assay according to the manufacturer's instructions (6016731, PerkinElmer) and the results were expressed as the fold change compared with the control.

For clonogenic survival assay, a total of 500 cells were seeded in 60-mm dishes coated with Gelatin (V900863, sigma) for 30 days (fresh medium was replaced every week). Colonies were stained with Coomassie brilliant blue solution R-250 (6104-59-2, BBI Life sciences), and then photographed for counting (> 50 cells in a colony). The results were expressed as the number of colony formation.

### Immunofluorescent (IF) staining

Cells were fixed with 4% formaldehyde for 15 min and then treated with 0.5% TritonX-100 for 10 min. Next, the cells were blocked for 30 min, and stained with anti-DEPTOR antibody (1:500) or anti-ErbB2 antibody (1:500, Santa Cruz) for 1 hr, followed by staining with secondary antibodies conjugated with Alexa Fluor 488 and 546 (1:500, Invitrogen) for 30 min and DAPI (1:500, Beyotime) for 30 min at room temperature. The cells were then photographed under a fluorescence microscope (Nikon).

### Human breast tissue microarray and immunohistochemistry (IHC)

Human breast tissue microarrays consisting of 67 pairs of tumors and adjacent normal tissues, and another 71 tumor samples from breast cancer patients were obtained from Outdo Biotech Company (Shanghai, China). The arrays were stained with anti-DEPTOR antibody and followed by quantitative evaluation as previously described 12. Briefly, the tissue microarrays were stained with anti-DEPTOR antibodies, followed by counterstaining with hematoxylin. The slides were then scanned by an Aperio Whole Slide Scanner. At least 5 random fields of each sample were photographed for calculating IHC score used to express the levels of DEPTOR.

### Quantitative RT-PCR

The quantitative RT-PCR analysis was performed as described previously 13. Briefly, total RNA was extracted from cells using TRIzol reagent (15596018, Invitrogen). cDNA was synthetized from RNA via reverse transcription using the PrimeScript RT reagent kit (RR037A, Takara). Quantitative real-time PCR was performed using SYBR Premix Ex Taq (RR420A, TaKaRa) on an Applied Biosystems StepOnePlus^TM^ Real-Time PCR instrument. The primer sequences were used as follows: ErbB2-F: 5'- CAG CCC CCA GCC TGA ATA T -3'; ErbB2-R: 5'- GCC GTA GGT GTC CCT TTG AA-3'; GAPDH-F: 5'-AGG GCA TCC TGG GCT ACA C-3'; and GAPDH-R: 5'- GCC AAA TTC GTT GTC ATA CCA G -3'.

### Flow cytometry

Cell were washed with ice-cold PBS and collected with 0.05% trypsin (Gibco), followed by centrifugation at 1000 g for 5 min. Cell pellets were then washed twice with PBS and stained with the Annexin V-FITC apoptosis detection kit (C1062, Beyotime) in the dark at room temperature for 15 min, followed by flow cytometry. All FACS data were analyzed with CytExpert software.

### Statistical analysis

The data from three independent experiments were expressed as the mean ± SEM and statistically analyzed by GraphPad Prism 8. The Wilcoxon rank sum test was utilized to analyze the DEPTOR expression in tumors and corresponding tumor-adjacent tissues. Kaplan-Meier survival curves were generated and compared using the log-rank test. The comparisons of parameters between two groups were determined by two-tailed Student's t test and the comparisons of parameters from more than two groups were determined by ANOVA. *p* < 0.05 was considered as statistically significant.

## Results

### DEPTOR overexpression in breast cancer tissues correlates with poor patient survival

**Figure 1:**
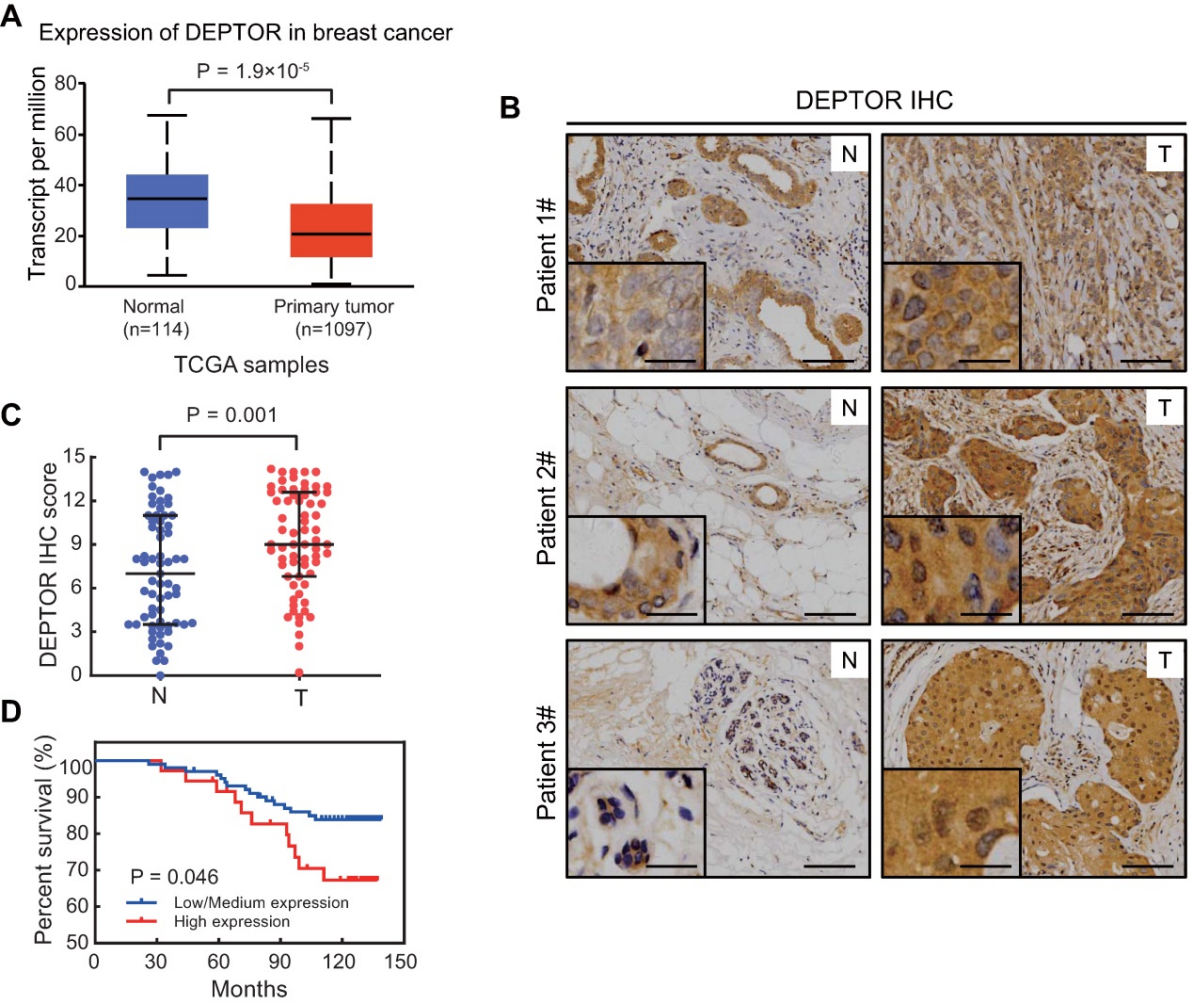
** DEPTOR expression is increased in breast tumor tissues and its overexpression correlates with poor patient survival.** A) The mRNA of DEPTOR is significantly reduced in primary breast tumors compared with normal tissues from TCGA database. *p* < 0.001. B-C. The protein level of DEPTOR is significantly increased in human breast cancer tissues. Breast tissue microarrays containing 67 tumor tissues (T) and their corresponding tumor-adjacent normal tissues (N) were stained for DEPTOR expression. Representative images of DEPTOR staining are shown (B) (scale bars: 100 µm) with a better resolution at the lower left corner (scale bars: 30 µm). The protein levels of DEPTOR are expressed as the immunoreactive score (IRS) calculated by multiplying the percentage score by the intensity score (C). The Wilcoxon rank sum test was utilized to assess the DEPTOR expression from 67 pairs of breast tissue samples. *p* = 0.001. D. High DEPTOR levels correlate with poor survival of patients with breast cancer. The cut-off value for high (*n* = 36) and low/medium (*n* = 102) expression of DEPTOR was determined with the Q75 of the immunoreactive score. Then, log-rank test between the two groups was performed and the corresponding Kaplan-Meier curves were generated (*p* = 0.046).

DEPTOR, acting as a direct inhibitor of mTOR, plays a key role in tumorigenesis 1, 6, 7. To clarify its role in breast tumorigenesis, we first examined DEPTOR mRNA expression using TPM (transcripts per million) data obtained from TCGA database, consisting of 1097 breast tumor and 114 normal breast tissues. As shown in Figure 1A, the mRNA level of DEPTOR was significantly decreased in breast cancer tissues compared to that in normal breast tissues (*p* < 0.001). Next, we examined potential alterations in DEPTOR protein expression in breast tumor tissues compared to adjacent normal tissues. We performed immunostaining on breast tissue microarrays, consisting of 67 pairs of tumor and corresponding tumor-adjacent normal tissues, using a DEPTOR antibody with specificity suitable for IHC staining 12. According to the staining intensity and the percentage of parenchymal cells with positive staining, breast tissues were classified into four groups (0, negative; 1, weak; 2, moderate; and 3, strong staining) and five groups (0, 0%; 1, ≤10%; 2, 11-50%; 3, 51-80%; and 4, ≥81%), respectively. DEPTOR protein levels were expressed as the IHC score, calculated by multiplying the percentage score by the intensity score. Surprisingly, we found that in 43 out of the 67 paired cases (64%), DEPTOR expression was higher in tumor tissues than in their corresponding tumor-adjacent normal tissues (Figure 1B), in contrast to the alterations in DEPTOR mRNA levels. The statistical analysis of the IHC scores performed by Wilcoxon rank sum test showed that DEPTOR expression was significantly increased in breast cancer tissues compared to their corresponding tumor-adjacent normal tissues (*p* = 0.001) (Figure 1C). The paradoxical alterations of DEPTOR expression between the mRNA and protein levels might be attributable to the post-translational modifications of the protein, particularly ubiquitination by SCF^β-TrCP^ and CUL5 ubiquitin ligases 14-17. Next, we determined the correlation between DEPTOR expression and patient survival using breast tissue microarrays, which consisted of 138 breast carcinomas, including 53 luminal A, 48 luminal B, 15 HER2 over-expression, and 22 basal-like. Based on the IHC scores of DEPTOR expression, we divided the patients into two groups: a high expression group with scores greater than the cut-off value Q75 (where Q75 is the 75^th^ percentile expression value), and a low/medium expression group with scores lower than Q75. The survival curves showed that patients showing a high expression of DEPTOR (*n* = 36) had worse overall survival than those showing a low or medium expression of DEPTOR (*n* = 102, *p* = 0.046, log-rank test, Figure 1D). Taken together, DEPTOR expression is increased in human breast cancer tissues and may serve as a biomarker for the prognosis of breast cancer patients, suggesting that the increase in DEPTOR expression might play a role in the development of human breast cancer.

### DEPTOR binds to ErbB2 on the cell membrane

**Figure 2:**
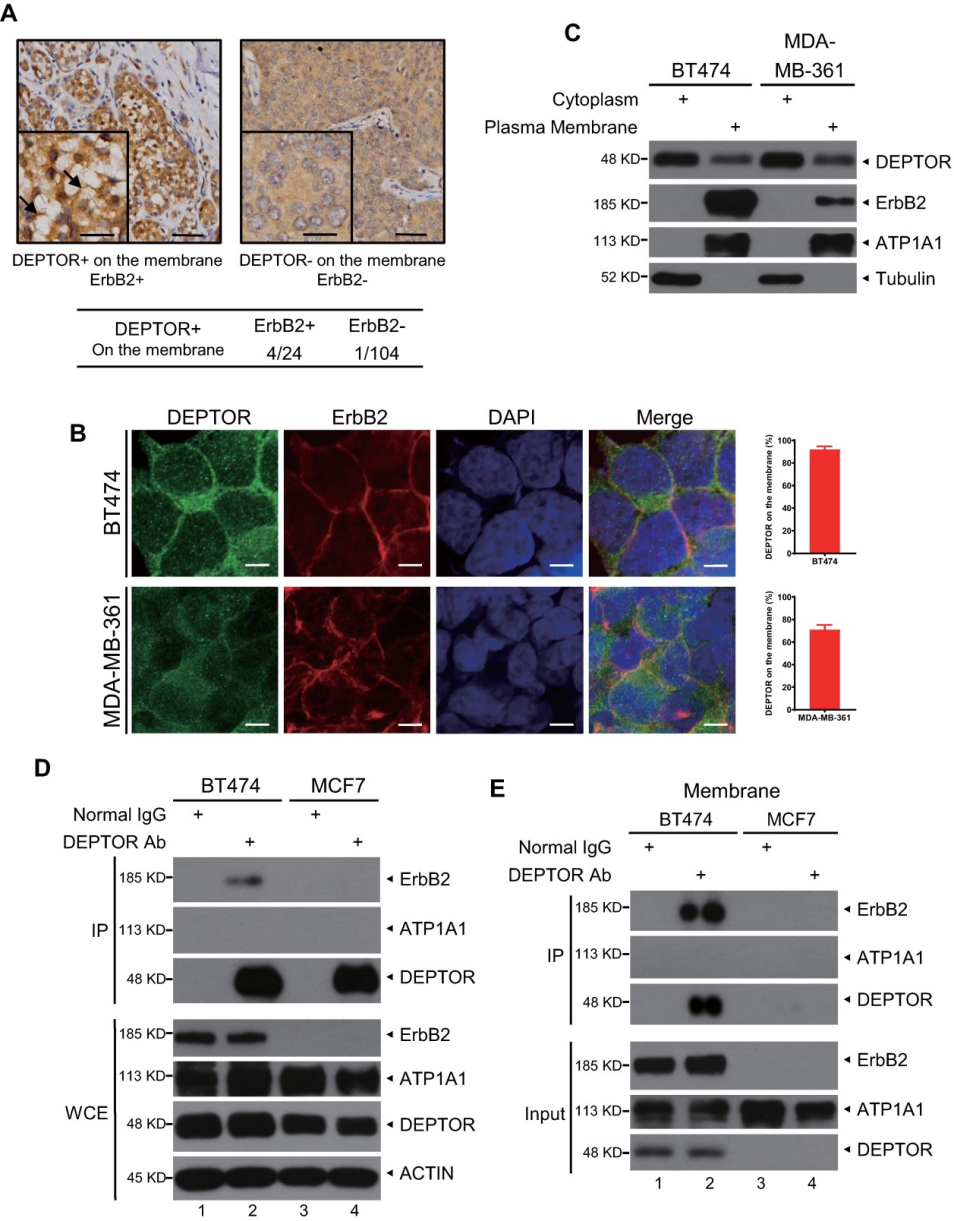
** DEPTOR is present on the cell membrane and binds to ErbB2 in ErbB2-positive breast cancer tissues and cells.** A) DEPTOR is localized on the membrane in human breast cancer tissues. Breast cancer tissue microarrays containing 138 tumor tissues and 67 tumor-adjacent normal tissues were stained for DEPTOR expression, and representative images are shown. Arrows indicate DEPTOR expression on the membrane. Scale bars represent 50 µm. The lower left corner of each immunohistochemistry image shows a part of the image at a higher resolution. Scale bars represent 20 µm. B-C. Localization of DEPTOR in ErbB2-positive breast cancer cells. The ErbB2-positive breast cancer cell lines BT474 and MDA-MB-361 were stained with the indicated antibodies (Abs) and photographed under a confocal fluorescence microscope (B), or harvested for subcellular fractionation, and subsequent immunoblotting (IB) with the indicated Abs (C). Scale bars represent 10 µm. Cells showing plasma membrane localization of DEPTOR were counted, and their numbers are expressed as the percentage of DEPTOR localized on the plasma membrane. ATP1A1 and Tubulin were used as plasma membrane and cytoplasmic markers, respectively. D-E. Binding of endogenous DEPTOR to ErbB2. BT474 and MCF7 whole cell lysates (D) or membrane fractions (E) were harvested for immunoprecipitation with anti-DEPTOR Ab, along with normal IgG control, followed by IB with the indicated Abs. WCE: whole-cell extract.

Next, we determined the potential mechanisms by which DEPTOR facilitates the development of human breast cancer. Surprisingly, we found that, in addition to cytoplasmic and nuclear localization, DEPTOR is also located on the cellular membrane in several breast tumor tissues but not in the tumor-adjacent normal tissues. The percentage of tumors with cell membrane-localized DEPTOR was significantly higher in ErbB2-positive tissues (4/24, 16.68%) than in ErbB2-negative tissues (1/104, 0.96%) (Figure 2A), indicating that DEPTOR may interact with ErbB2 on the membrane. To determine the interplay between DEPTOR and ErbB2 in breast cancer cells, we first measured the protein levels of DEPTOR and ErbB2 in multiple breast cancer cells and found that both ErbB2-positive BT474 and MDA-MB-361 cells showed a high expression of DEPTOR and ErbB2, while ErbB2-negative MCF7 cells showed a moderate expression of DEPTOR (Figure S1A). Furthermore, the autophosphoarylation of ErbB2 is high in BT474 and MDA-MB-361 cells, indicating high kinase activity of ErbB2. Thus, we chose BT474 and MDA-MB-361 cells to explore the interplay between DEPTOR and ErbB2, and chose MCF7 cells as an ErbB2-negative control. To explore the localization of DEPTOR in breast cancer cells, we first examined the specificity of DEPTOR antibody in an IF assay using BT474 cells transfected with siRNA targeting DEPTOR (siDEPTOR). We found that DEPTOR-positive signals were significantly reduced in siDEPTOR cells compared with those in siGFP cells (Figure S1B), suggesting that the DEPTOR antibody has good specificity and is suitable for IF in human breast cancer cells. Next, we performed IF with this antibody and confirmed that endogenous DEPTOR was co-localized with ErbB2 on the membranes of ErbB2-positive BT474 and MDA-MB361 cells (Figure 2B), but not on the membranes of ErbB2-negative MCF7 cells (Figure S1C). DEPTOR was readily detected in the plasma membrane fractions of both BT474 and MDA-MB-361 cells, but not in the membrane fractions of ErbB2-negative ZR75-1 and MCF7 cells (Figures 2C and S1D). For the subcellular fractionation assays, we used ATP1A1 and Tubulin as markers of the membrane and cytoplasmic fractions, respectively, and the result showed our subcellular fractionation was performed successfully (Figures 2C and S1D). Finally, we examined the potential interaction between DEPTOR and ErbB2 and found that ErbB2 was readily detected in immunoprecipitants by DEPTOR from whole cell lysates, as well as membrane fractions of ErbB2-positive BT474 cells, but not of ErbB2-negative MCF7 cells (Figure 2D and 2E), and endogenous ErbB2 pulled down endogenous DEPTOR under physiological conditions (Figure S1E). ATP1A1, a typical membrane protein, was not detected in any immunoprecipitant, implying the specificity of the interaction between DEPTOR and ErbB2. Taken together, these results indicate that DEPTOR is anchored on the cell membrane, where it binds to ErbB2.

### DEPTOR binds to ErbB2 *via* its PDZ domain

**Figure 3:**
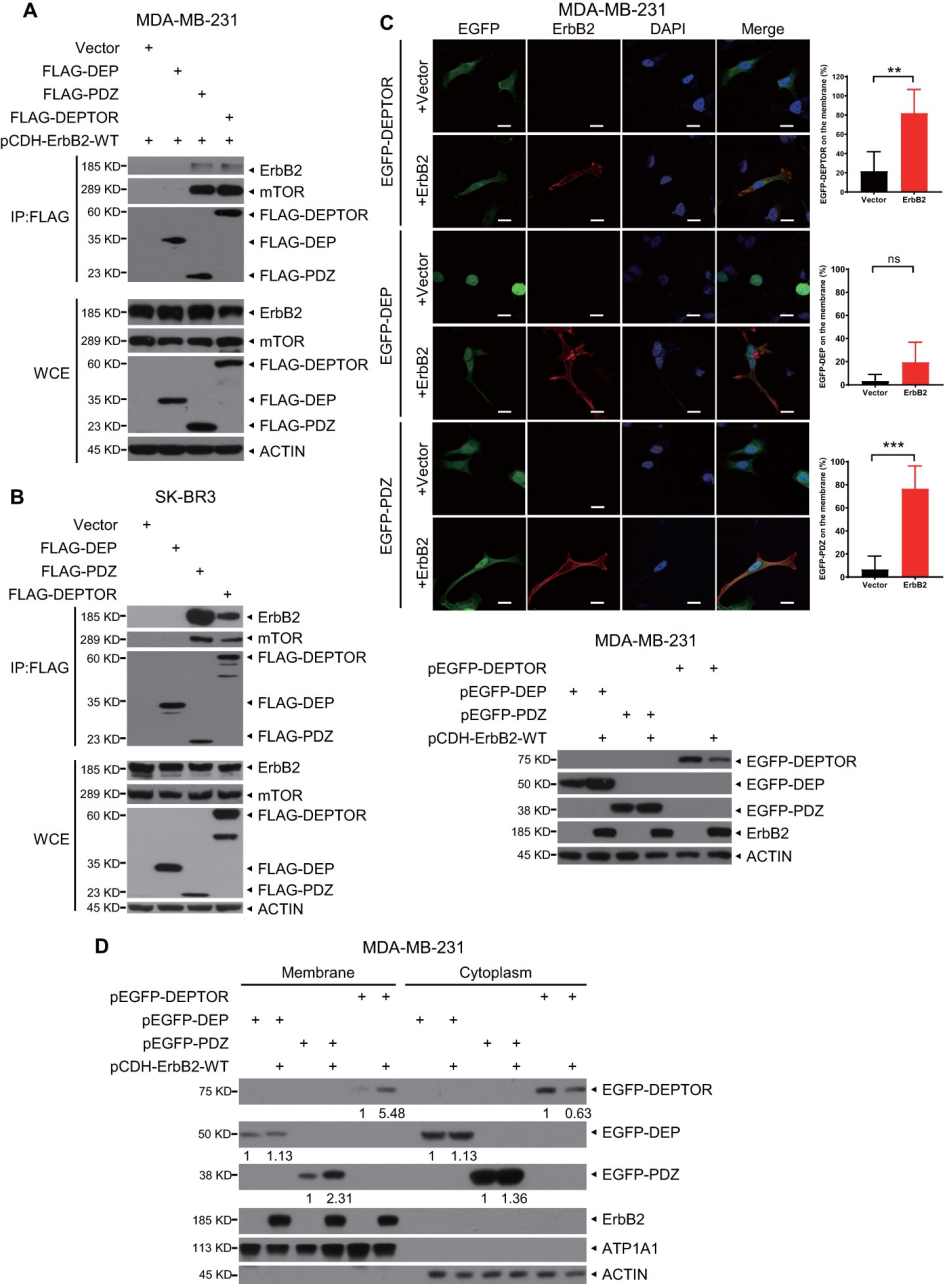
** The PDZ domain of DEPTOR binds to ErbB2 and facilitates the membrane localization of DEPTOR.** A-B. DEPTOR binds to ErbB2 *via* its PDZ domain. MDA-MB-231 and SK-BR3 cells were transfected with the indicated plasmids, followed by immunoprecipitation with an anti-FLAG antibody (Ab) and then immunoblotting (IB) with the indicated Abs. C-D. The PDZ domain facilitates anchorage of DEPTOR to the cell membrane. MDA-MB-231 cells were transfected with the indicated plasmids for 48 hrs, and then subjected to immunofluorescence staining (up, C), IB (bottom, C), or subcellular fractionation (D). Scale bars represent 20 µm. Cells showing the plasma membrane localization of DEPTOR or its domains were counted, and their numbers are expressed as the percentage of DEPTOR, or its domains, localized on the plasma membrane.

DEPTOR comprises two N-terminal tandem DEP domains and a C-terminal PDZ domain. To determine which domain mediates its interaction with ErbB2, we co-transfected an ErbB2-expressing construct with a construct expressing FLAG-tagged DEP domains, PDZ domain, or full-length DEPTOR, and then performed an IP assay. We found that both full-length DEPTOR and its PDZ domain, but not its DEP domains, pulled down ectopically expressed or endogenous ErbB2 as well as endogenous mTOR (as a positive control; Figures 3A, 3B, and S2A). To determine whether ErbB2 regulates the subcellular localization of DEPTOR, we co-transfected ErbB2 with EGFP-fusions of full-length DEPTOR or its domains into ErbB2-negative breast cancer MDA-MB-231 cells or HEK293 cells. Interestingly, EGFP-fused full-length DEPTOR was uniformly located in the cytoplasm and nucleus, whereas the EGFP-fused DEP domain was mostly trapped in the nucleus (Figures 3C and S2B). Notably, overexpression of ErbB2 significantly increased the membrane localization of full-length DEPTOR and its PDZ domain (Figures 3C and S2B). Additionally, a subcellular fractionation assay confirmed that ErbB2 overexpression increased the levels of full-length DEPTOR and its PDZ domain by 5.48- and 2.31-fold, respectively, on the membrane of MDA-MB-231 cells, and 4.12- and 3.38-fold, respectively, on the membrane of HEK293 cells (Figures 3D and S2C). Although the DEP domain was also detected in the membrane fraction, its levels in the membrane fraction did not change in MDA-MB-231 cells and were decreased by 0.47-fold in HEK293 cells when ErbB2 was co-transfected (Figures 3D and S2C). Taken together, these results show that ErbB2 promotes cell membrane localization of DEPTOR by binding to its PDZ domain.

### DEPTOR competes with β-TrCP to bind and stabilize ErbB2

**Figure 4:**
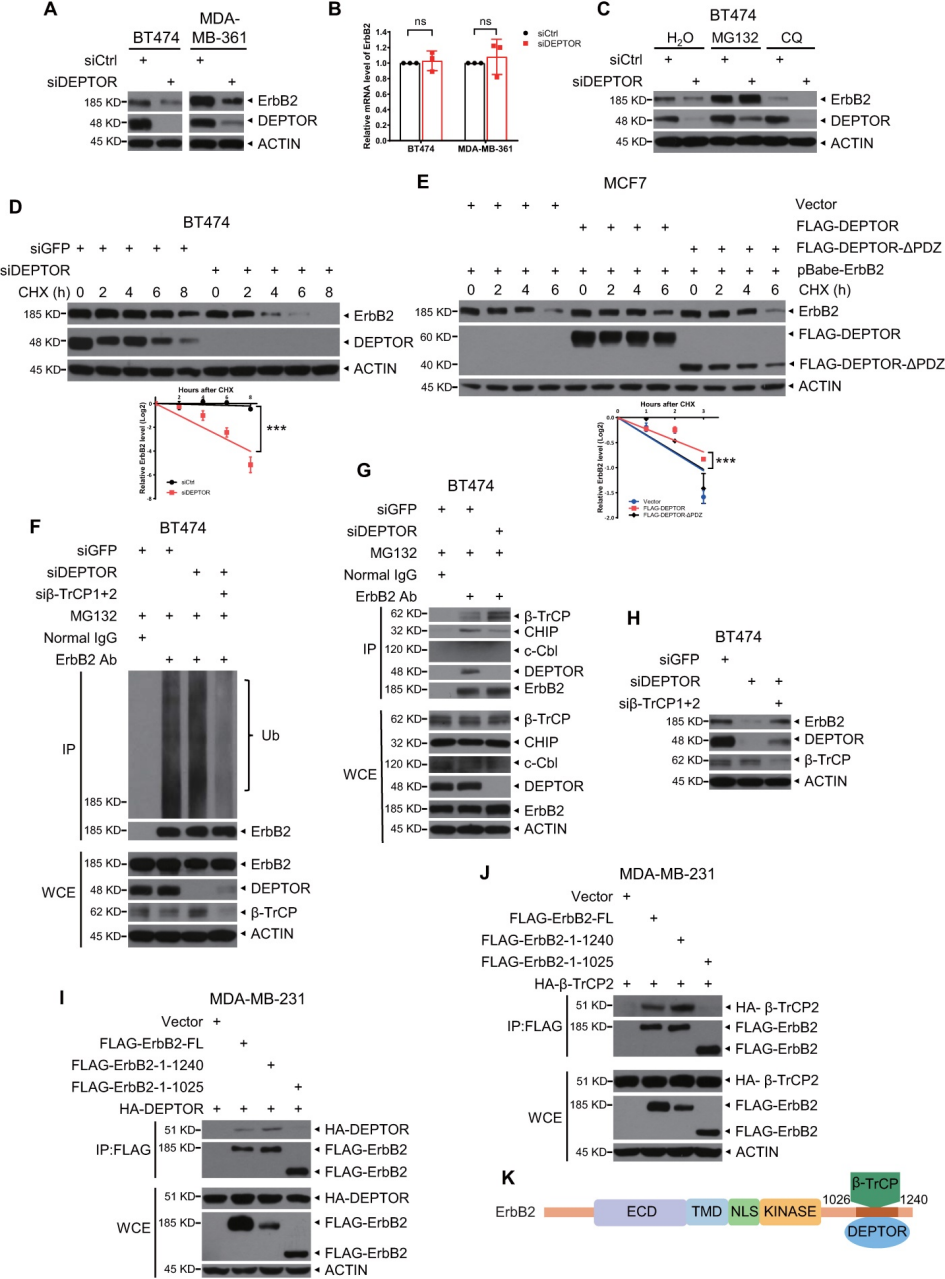
** DEPTOR stabilizes ErbB2 by competitively inhibiting ErbB2-β-TrCP binding.** A-B. DEPTOR knockdown reduces ErbB2 levels. BT474 and MDA-MB-361 cells were transfected with siRNA targeting DEPTOR or scrambled control siRNA (siCtrl), followed by IB (A) or qRT-PCR (B, n = 3). C-G. DEPTOR silencing destabilizes ErbB2 by proteasome pathway instead of lysosomal pathway and DEPTOR overexpression stabilizes ErbB2: BT474 cells were transfected siDEPTOR or siCtrl for 48 hrs and then incubated with MG132 for 5 hrs or CQ for 12 hrs (C), or CHX for indicated time periods (D), followed by IB with indicated Abs. MCF7 cells infected with retrovirus stably expressing ErbB2 were transfected with indicated plasmids for 48 hrs and then incubated with CHX for indicated time periods (E), followed by IB with indicated Abs. Densitometry quantification was performed with Image J, and the decay curves are shown (mean ± S.E.M., n = 3, ***p < 0.001) (bottom, D and E). BT474 cells infected with lentivirus expressing indicated shRNA were incubated with MG132 for 5 hrs and then subjected to IP with anti-ErbB2 Ab, along with normal IgG control, followed by IB with indicated Abs (F-G). G. DEPTOR knockdown enhances the interaction of ErbB2 and β-TrCP. H. Simultaneous silencing of DEPTOR and β-TrCP abrogates ErbB2 reduction by DEPTOR knockdown. BT474 cells were infected with lentivirus expressing indicated shRNA for 48 hrs, followed by IB with indicated Abs. I-K. Both DEPTOR and β-TrCP2 interact with 1026-1240 amino acids of ErbB2: MDA-MB-231 cells were transfected with the indicated plasmids, followed by IP with an anti-FLAG Ab and then IB with the indicated Abs (I-J). K. A model for competitive binding of DEPTOR and β-TrCP to the same region of ErbB2.

Next, we determined the effects of DEPTOR binding to ErbB2. First, we found that silencing of DEPTOR significantly decreased the protein levels of ErbB2 in BT474 and MDA-MB-361 cells (Figure 4A) without affecting its mRNA levels (Figure 4B), suggesting that the reduction in ErbB2 levels caused by DEPTOR knockdown may occur at the post-translational level. Indeed, the proteasomal inhibitor MG132, but not the lysosomal inhibitor chloroquine, completely rescued the decrease in ErbB2 levels caused by DEPTOR knockdown, indicating that DEPTOR knockdown destabilizes ErbB2 *via* the proteasome pathway, but not *via* the lysosomal pathway (Figure 4C). On the other hand, DEPTOR overexpression increased the protein levels of ectopically expressed ErbB2 in MCF7 and T47D cells (Figure S3A). Consistently, using cycloheximide to block new protein synthesis, we found that the half-life of ErbB2 protein was evidently shortened following DEPTOR depletion (Figure 4D), and the overexpression of full-length DEPTOR, but not of a mutant with PDZ domain deleted (DEPTOR-∆PDZ), significantly extended the protein half-life of ErbB2 ectopically expressed in MCF7 cells (Figure 4E). To exclude the effect of mTOR regulation on ErbB2 stability, we treated siGFP and siDEPTOR cells with rapamycin, an inhibitor of mTORC1, and found that DEPTOR knockdown significantly shortened the protein half-life of ErbB2 with rapamycin (Figure S3B, lanes 13-16 vs. 9-12), suggesting the direct role of DEPTOR on ErbB2 degradation. Interestingly, we noticed that in DEPTOR knockdown cells, the protein half-life of ErbB2 was moderately extended under rapamycin treatment, compared that with DMSO treatment (Figure S3B, lanes 13-16 vs. 5-8). This is likely due to the inhibition of DEPTOR degradation by rapamycin, which is well characterized 14. Consistently, the polyubiquitination of endogenous ErbB2 was also increased in DEPTOR-knockdown cells (Figure 4F, lanes 3 vs. 2). Thus, these results suggest that DEPTOR knockdown promotes the proteasomal degradation of ErbB2. It has been shown that ErbB2 is targeted for ubiquitination by several E3 ubiquitin ligases, including β-TrCP, CHIP, and c-Cbl, leading to its proteasomal degradation 18-21. To identify the ligase that promotes ErbB2 degradation upon DEPTOR depletion, we performed co-IP experiments and found that DEPTOR silencing decreased the levels of CHIP pulled down by ErbB2, while increasing the levels of β-TrCP pulled down by ErbB2, suggesting possible disruption of ErbB2-β-TrCP binding by DEPTOR (Figure 4G). Interestingly, c-Cbl was undetectable in the immunoprecipitants and whole cell lysates. Importantly, the simultaneous silencing of β-TrCP abrogated the reduction in the levels of ErbB2 caused by DEPTOR knockdown (Figure 4H), and decreased the polyubiquitination of endogenous ErbB2 (Figure 4F, lanes 4 vs. 2-3), suggesting a causal role of β-TrCP in ErbB2 degradation upon DEPTOR knockdown. It has been reported that PTPN18 interacts with ErbB2 at the pY1112 site and recruits β-TrCP to mediate ErbB2 ubiquitination 18. To determine the interplay between DEPTOR, ErbB2, and β-TrCP, we generated two truncated mutants as follows: 1) Mutant-1/ErbB2_1-1240_ with deletion of the PDZ domain (D_1252_-V_1255_), which mediates ErbB2 binding to the PDZ domain of Erbin 22; and 2) mutant-2/ErbB2_1-1025_ with both deletions of the Y1112 residue and PDZ domain. We found that both full-length ErbB2 and mutant-1/ErbB2_1-1240_, but not mutant-2/ErbB2_1-1025_, pulled down either DEPTOR (Figure 4I) or β-TrCP2 (Figure 4J), indicating that DEPTOR and β-TrCP competitively bind to the residues 1026 to 1240 of ErbB2 (Figure 4K). Taken together, these results suggest that DEPTOR competes with β-TrCP for ErbB2 binding, thus inhibiting β-TrCP-mediated ErbB2 ubiquitination and degradation.

### DEPTOR knockdown represses cell proliferation and survival by inducing apoptosis

**Figure 5:**
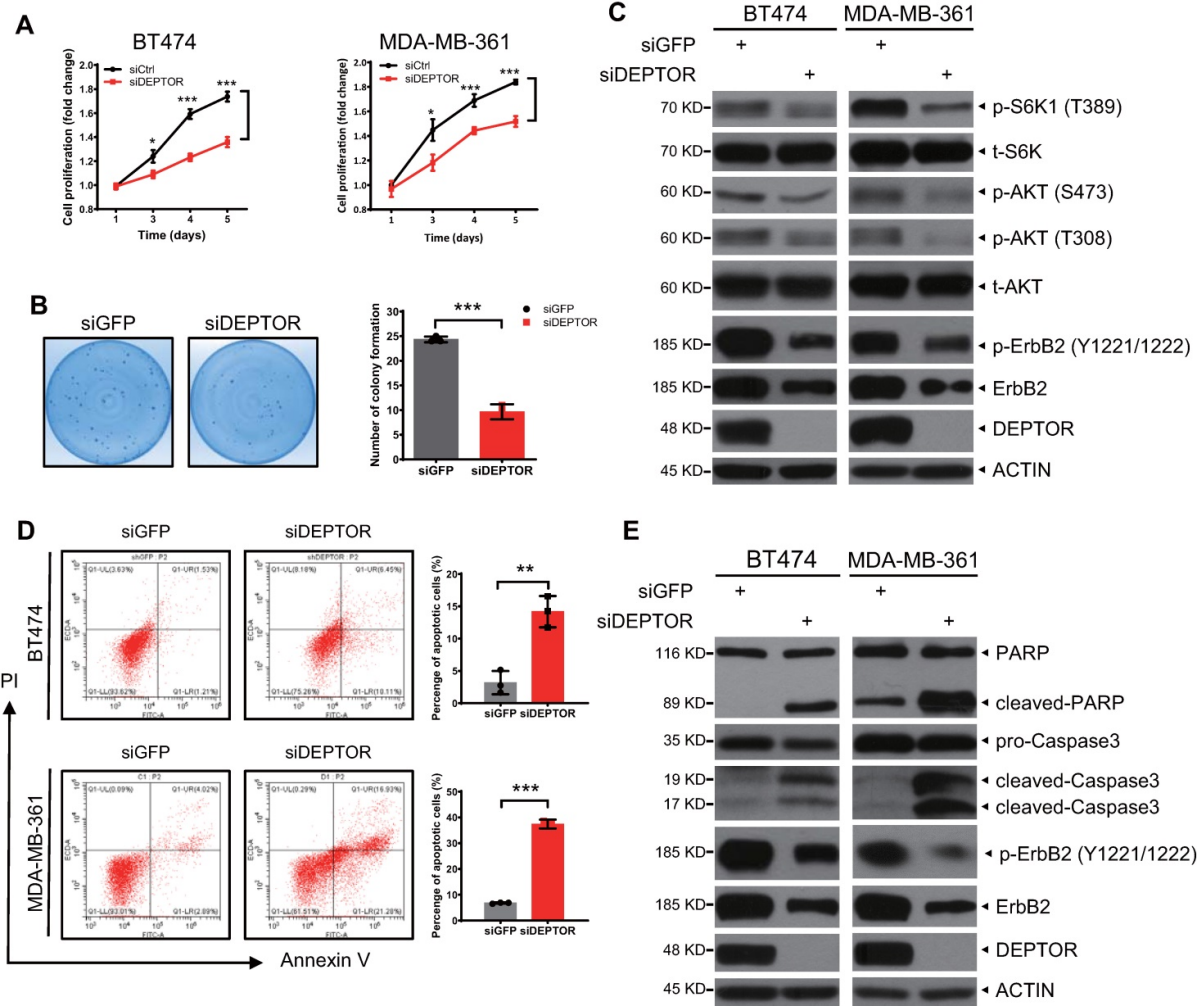
** DEPTOR knockdown suppresses cell proliferation and survival and promotes apoptosis by inhibiting PI3K/AKT/mTOR signaling.** A-B. DEPTOR knockdown suppresses cell proliferation and survival. BT474 and MDA-MB-361 cells were transfected with indicated siRNA (A) or infected with lentivirus-based shRNA (B) as indicated, followed by ATPlite cell proliferation assay (A) and clonogenic survival assay (B). C. DEPTOR silencing inactivates PI3K/AKT/mTOR signaling. Cells infected with lentivirus-based shRNA were harvested for IB with indicated Abs. D-E. DEPTOR knockdown induces apoptosis. Cells infected with lentivirus-based shRNA were subjected to flow cytometry using the Annexin V-FITC apoptosis detection kit (D) and IB with indicated Abs (E). Shown are mean ± SEM from three independent experiments, n = 3; *p < 0.05; **p < 0.01; ***p < 0.001.

We further determined the biological significance of ErbB2 depletion triggered by DEPTOR knockdown. We found that DEPTOR knockdown by two different targeting sequences significantly suppressed the proliferation of ErbB2-positive cells (Figures 5A and S5B) and dramatically decreased colony formation, indicating a reduction in cell survival (Figures 5B and S5C). Mechanistically, given that DEPTOR is a natural inhibitor of mTORC1/2 complexes, we next determined whether DEPTOR depletion resulted in the activation of S6K1 and AKT, the downstream effectors of mTORC1 and mTORC2, respectively. Unexpectedly, the phosphorylation of S6K1 and AKT was decreased in DEPTOR-knockdown cells (Figures 5C and S5E). It is well known that AKT is phosphorylated at Ser473 and Thr308 by mTORC2 and PI3K/PDK1 activated by ErbB2, respectively, and that activated AKT promotes mTORC1 activation 8. The decrease in S6K1 and AKT phosphorylation upon DEPTOR silencing suggested that the reduction in the levels of ErbB2 by DEPTOR knockdown appears to play a major role in suppressing the PI3K/AKT/mTOR pathway in ErbB2-positive breast cancer cells. Consistent with this, the percentage of apoptotic cells, as determined by flow cytometric analysis of Annexin V^+^ cells, was significantly increased upon DEPTOR knockdown and the subsequent ErbB2 reduction (Figures 5D and S5D). Likewise, DEPTOR knockdown markedly increased the levels of cleaved PARP and caspase 3, two markers of apoptosis (Figures 5E and S5E). Interestingly, in ErbB2-negative MCF7 cells, DEPTOR knockdown had no significant effect on cell proliferation, survival, and apoptosis (Figure S4A-C). Meanwhile, the immunoblotting results confirmed that DEPTOR knockdown had minor, if any, effect on the phosphorylation of S6K1 and AKT in MCF7 cells (Figure S4D). Taken together, our results suggest that DEPTOR knockdown suppresses cell proliferation and survival by inactivating the PI3K/AKT/mTOR pathway to induce apoptosis in ErbB2-positive breast cancer cells.

### Constitutively active ErbB2-YVMA rescues the reduction in cell proliferation and survival induced by DEPTOR depletion

**Figure 6:**
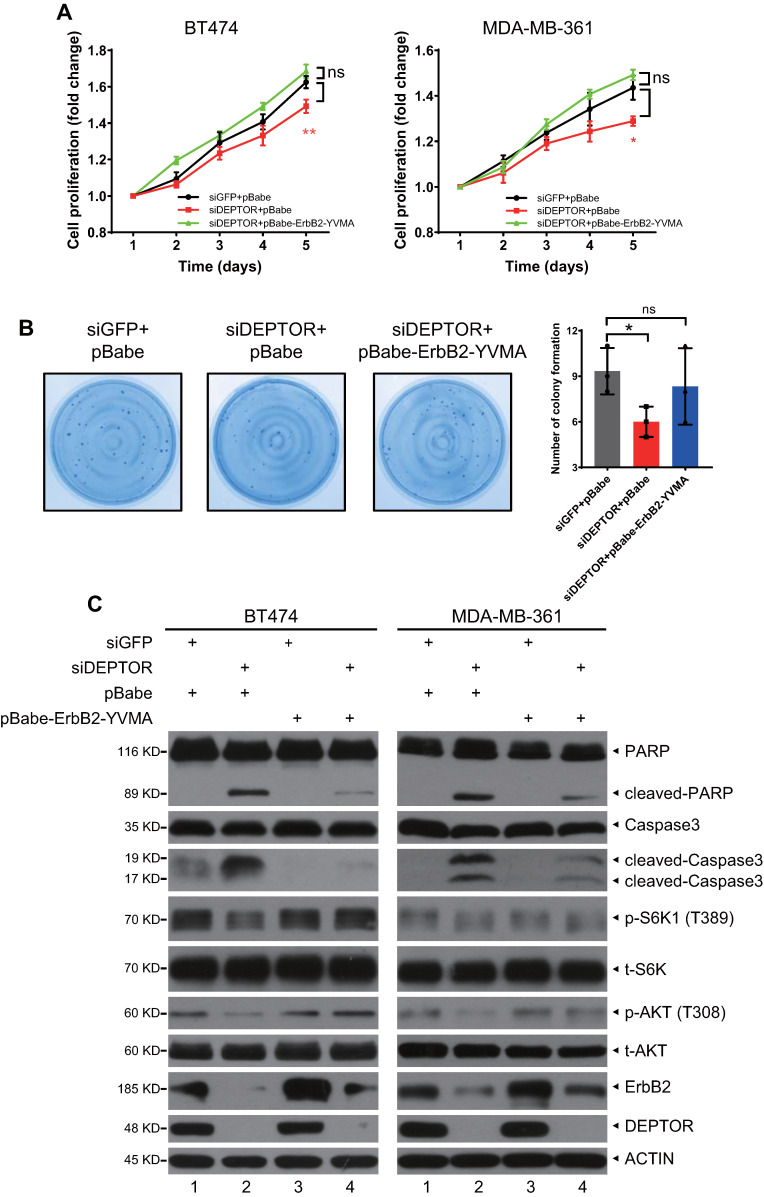
** Ectopic expression of constitutively active mutant ErbB2-YVMA rescues the suppression of cell proliferation and survival by DEPTOR depletion.** A-C. BT474 and MDA-MB-361 cells infected with lentivirus-based shRNA were infected with retrovirus expressing ErbB2-YVMA or mock vector and then subjected to puromycin selection. Stable clones were pooled for ATPlite cell proliferation assay (A), clonogenic survival assay (B), and IB with indicated Abs (C). Shown are mean ± SEM from three independent experiments, n = 3; ns, not significant; *p < 0.05; **p < 0.01.

To determine whether ErbB2 degradation triggered by DEPTOR knockdown plays a causal role in the inhibition of cell proliferation and survival, we ectopically expressed ErbB2-YVMA, a constitutively active ErbB2 A775-G776insYVMA mutant from human tumor patients 23, in DEPTOR-depleted cells. Given that this mutation has only four residues between A775 and G776 and would have no effect on ErbB2-DEPTOR interaction and its membrane localization. Although the expression of ErbB2-YVMA was lower than that of wild-type ErbB2, it significantly activated the downstream signaling pathways of ErbB2, as reflected by the increased phosphorylation of ERK1/2, S6K1, and AKT (Figure S5A, lane 3 vs. 2). Indeed, the ectopic expression of ErbB2-YVMA not only completely abrogated the reduction in cell proliferation by DEPTOR knockdown, but also slightly increased the proliferation of BT474 and MDA-MB-361 cells, when compared with the corresponding levels of the control cells (Figure 6A). ErbB2-YVMA also reversed the reduction in cell survival by DEPTOR knockdown in BT474 cells (Figure 6B). Likewise, ErbB2-YVMA reversed the reduction in PI3K/AKT/mTOR pathway activity, as reflected by the increased phosphorylation of S6K1 and AKT (Figure 6C, lanes 4 vs. 2). In addition, induction of apoptosis by DEPTOR knockdown was inhibited by ErbB2-YVMA, as reflected by the decrease in the levels of cleaved PARP and caspase 3 (Figure 6C, lanes 4 vs. 2).

Taken together, these results demonstrate that the reduction in ErbB2 plays a causal role in the suppression of cell proliferation and survival and the induction of apoptosis upon DEPTOR depletion.

We further determined whether the simultaneous knockdown of β-TrCP would reverse the changes in cell proliferation and apoptosis by DEPTOR knockdown. Interestingly, the simultaneous β-TrCP knockdown did not reverse the inhibition of cell proliferation and induction of apoptosis by DEPTOR knockdown, but contrarily further enhanced these changes. Mechanistically, although simultaneous β-TrCP knockdown restored the expression of ErbB2 and activity of mTORC1 (Figure S5E, lanes 3 vs. 2), as reflected by increased phosphorylation of S6K1, the obvious induction of apoptosis, as reflected by significant induction of Annexin V+ cells (Figure S5D) and cleavage of PARP and caspase-3 (Figure S5E, lanes 3 vs. 2), might be attributable to the dramatic increase in the levels of BIMEL (Figure S5E, lanes 3 vs. 2), a pro-apoptotic protein and well-known substrate of β-TrCP 24, by an unknown mechanism.

## Discussion

In this study, we demonstrated that DEPTOR functions as a positive regulator of ErbB2 in ErbB2-positive breast cancer cells, which may contribute to the development of human breast cancer. Our conclusions are supported by the following lines of evidence: (1) DEPTOR expression is increased in human breast cancer tissues, and its overexpression correlates with poor patient survival; (2) DEPTOR is expressed on the cell membrane of ErbB2-positive breast cancer tissues and cells; (3) DEPTOR binds to ErbB2 on the cell membrane *via* its PDZ domain; (4) DEPTOR disrupts the interaction between ErbB2 and its E3 ligase β-TrCP, thus stabilizing ErbB2; (5) DEPTOR depletion promotes ErbB2 degradation *via* the proteasome pathway to inactivate the PI3K/AKT/mTOR pathway and subsequently induce apoptosis, thereby reducing cell proliferation and survival; and (6) a constitutively active ErbB2 mutant (ErbB2-YVMA) completely abrogates the effects of the reduction in ErbB2 levels by DEPTOR depletion.

DEPTOR, as a natural inhibitor of mTOR, blocks the activities of both mTORC1 and mTORC2 by directly binding to mTOR, and functions as a tumor suppressor 1, 5, 12. However, under certain circumstances, DEPTOR can act as an oncoprotein by relieving feedback inhibition of PI3K, resulting in activation of AKT 1, 5, 6. Under physiological conditions, DEPTOR is mainly distributed in the cytoplasm. In this study, we made a novel observation that DEPTOR translocates to the cell membrane in ErbB2-positive breast tumor tissues, but not in tumor-adjacent normal tissues, and its PDZ domain is responsible for ErbB2 binding. More importantly, ErbB2 facilitates the localization of DEPTOR to the cell membrane, suggesting that high levels of ErbB2 may play a role in recruiting DEPTOR to the membrane (Figures 2 and 3). Furthermore, recruitment of DEPTOR by ErbB2 further stabilizes ErbB2 and increases its phosphorylation (Figure S3A). However, the increased level of ErbB2 phosphorylation is likely due to overall induction of ErbB2 protein levels, since the binding regions of DEPTOR on ErbB2 is not located in its kinase domain. Importantly, the interaction of DEPTOR with ErbB2 enhances the functions of ErbB2 in cell proliferation and survival (Figures 4 and 5), which implies that DEPTOR might promote ErbB2-mediated breast tumorigenesis and have oncogenic characteristics. Interestingly, the fusion genes identified by analyzing breast cancer genome rearrangements indicated that ErbB2 might serve as an adaptor for DEPTOR in regulating the mTOR pathway 25, thus adding another layer of complexity in the cross-talk between DEPTOR and ErbB2 in breast tumorigenesis. It has been shown that DEPTOR expression is decreased in aggressive breast cancers, and paradoxically, it inhibits breast cancer growth and invasion 26. Together with our findings, the results of these studies demonstrate the complexity of the role of DEPTOR in the regulation of breast tumorigenesis. Given that DEPTOR can act as either a tumor suppressor or an oncoprotein in cell culture settings, it is necessary to examine the role of DEPTOR in breast tumorigenesis *in vivo*, particularly in *Deptor-*knockout (KO) mouse models. Specifically, as *Deptor* whole body KO mice (*Deptor^-/-^*) develop normally and exhibit no obvious phenotypes 12, 27, it would be appropriate to study whether *Deptor^-/-^* or conditional KO in the breast delays breast tumorigenesis triggered by activated rat ErbB2 under the control of the mouse mammary tumor virus (MMTV) long terminal repeat (LTR) promoter (*MMTV-ErbB2*), which specifically expresses activated ErbB2 in the mammary gland epithelium.

It has been shown that DEPTOR is overexpressed in multiple myeloma cells and is required for cell survival. DEPTOR depletion significantly blocks AKT signaling, in part by decreasing the levels of PDGFR-β, a cell surface tyrosine kinase receptor, and also impairs PI3K-AKT activation by decreasing IRS1 protein levels 5. Several other studies have also shown that DEPTOR accumulation drives AKT activation by relieving feedback inhibition from S6K1 to PI3K, which confers cell survival in the muscle 28 and tumor cells 1, 14, 29. Our study also showed that DEPTOR is indispensable for the proliferation and survival of ErbB2-positive breast cancer cells (Figure 5). We further revealed that DEPTOR translocates to the membrane to prevent β-TrCP-mediated ubiquitination and degradation of ErbB2 (Figure 2-4), thereby activating AKT signaling. Thus, our study adds another layer of complexity to the process of DEPTOR activating AKT by stabilizing ErbB2. Interestingly, upon mTORC1 activation, one of the downstream signals of ErbB2, β-TrCP also mediates DEPTOR ubiquitination and degradation 14-16. Indeed, endogenous DEPTOR could pull down endogenous β-TrCP (Figure S5F), and β-TrCP knockdown caused DEPTOR accumulation (Figure S5E, lanes 4 vs. 1). Thus, a feedback loop may be established between DEPTOR, ErbB2, and β-TrCP.

What are the translational implications of this study? ErbB2 is overexpressed in 20-30% of breast cancers and is correlated with poor prognosis 9. It is also upregulated in several other cancers, such as colon, ovarian, pancreatic, and prostate cancers 30. In addition, ErbB2 has been shown to be an attractive target for ErbB2-positive breast cancer, and several ErbB2-targeting agents, such as trastuzumab, pertuzumab, and lapatinib, have been approved for the treatment of ErbB2-positive breast cancer by the U.S. Food and Drug Administration 31. Our finding that DEPTOR binds to and stabilizes ErbB2 on the cell membrane offers a new strategy for targeting ErbB2, which can be focused on the following two aspects. First, small molecular compounds can be developed to disrupt the binding of DEPTOR to ErbB2, as small molecule inhibitors of DEPTOR-mTOR interaction have been shown to be cytotoxic against multiple myeloma cells 32, 33. Second, given that DEPTOR is considered to be a potential therapeutic target in some cancers, such as multiple myeloma 33, our study also suggest that targeting DEPTOR to repress its expression through a variety of means, such as disturbing its transcription or promoting its degradation 5, 14-17, 28, 34, 35, and especially by developing proteolysis-targeting chimeric molecules that directly degrade DEPTOR 36, might have potential therapeutic value in ErbB2-overexpressing breast cancer.

**Figure 7:**
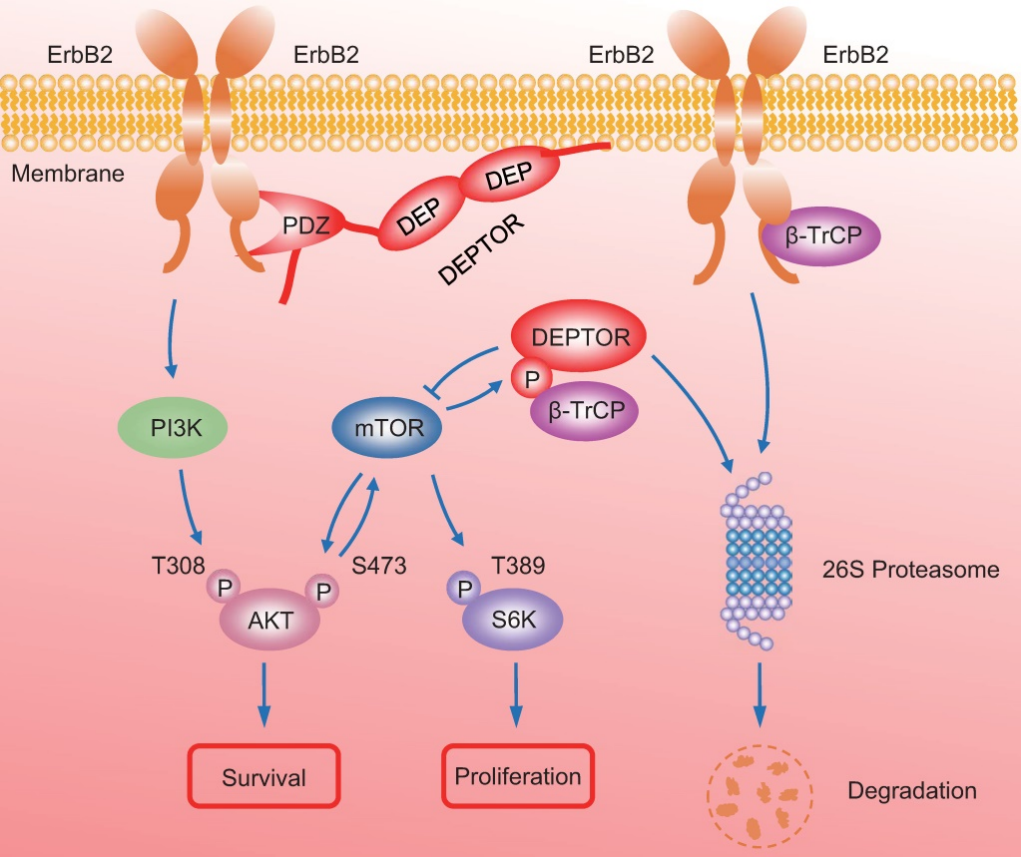
** A model for DEPTOR-ErbB2-β-TrCP interplay in regulation of cell proliferation and survival in ErbB2-positive breast cancer cells.** See text for details.

In summary, our study uncovered a crucial interplay between DEPTOR and ErbB2 in the regulation of cell proliferation and survival in ErbB2-positive breast cancer cells. DEPTOR translocates to the cell membrane and binds to ErbB2 (residues 1026 to 1240) *via* its PDZ domain, which prevents β-TrCP-mediated proteasomal degradation of ErbB2. Stabilized ErbB2 then relays extracellular signals to activate downstream intracellular signaling cascades, leading to the activation of AKT and S6K1 to promote cell proliferation and survival. Meanwhile, both mTOR and β-TrCP are negative regulators of DEPTOR, and involved in a negative-feedback regulatory loop by which activated mTORC1, a downstream signal of ErbB2, phosphorylates the β-TrCP binding motif of DEPTOR and mediates its ubiquitination and degradation by SCF^β-TrCP^, which then promotes the interaction of β-TrCP and ErbB2, and the subsequent ErbB2 degradation, ultimately leading to the inactivation of mTORC1. Upon DEPTOR knockdown, ErbB2 is degraded by the proteasome pathway to inactivate ErbB2/PI3K/AKT signaling, leading to the suppression of cell proliferation and survival. The balance between DEPTOR and ErbB2 plays an essential role in the maintenance of cellular homeostasis under ErbB2-positive conditions (Figure 7). Thus, the discovery of small molecules targeting the interaction between DEPTOR and ErbB2 might be an attractive approach for ErbB2-positive breast cancer therapy.

## Supplementary Material

Supplementary figures.

## Abbreviations

Ab: antibody
ATCC: American Type Culture Collection
CHX: cycloheximide
CQ: chloroquine
DEP: Disheveled, EGL-10, and Pleckstrin
DEPTOR: DEP domain containing mTOR interacting protein
DMEM: Dulbecco's modified Eagle's medium
ERBB: epidermal growth factor receptor
FBS: fetal bovine serum
IB: immunoblotting
IF: immunofluorescent
IHC: immunohistochemistry
IP: immunoprecipitation
LTR: long terminal repeat
MMTV: mouse mammary tumor virus
PDZ: PSD95, Dlg and ZO-1
TPM: transcripts per million
YVMA: Tyr, Val, Met and Ala
