# Supplementary material for: DEPTOR stabilizes ErbB2 to promote the proliferation and survival of ErbB2-positive breast cancer cells
Source: Theranostics. 2021 Apr 19;11(13):6355–69. doi: 10.7150/thno.51286 (PMC8120212; doi:10.7150/thno.51286)

Supplemental information

Figure S1. DEPTOR is present on the cell membrane and binds to ErbB2 in ErbB2-positive breast cancer cells

A. The expression of DEPTOR and ErbB2 in multiple breast cancer cell lines: Breast cancer cells were harvested for immunoblotting (IB) with the indicated antibodies (Abs). Human breast cancer BT474 and MDA-MB-361 cells with high expression of DEPTOR and ErbB2 and MCF7 cells with moderate expression of DEPTOR and low expression of ErbB2 were selected for this study. B. The specificity of DEPTOR antibody used in immunofluorescent (IF) staining: BT474 cells infected with lentivirus-based shGFP and shDEPTOR were stained using DEPTOR antibody. Scale bars represent 10 μm. C-D. Localization of DEPTOR in ErbB2-positive or -negative breast cancer cells. The breast cancer cells were stained with the indicated Abs and photographed under a confocal fluorescence microscope (C), or harvested for subcellular fractionation, and subsequent IB with the indicated Abs (D). Scale bars represent 20 μm. ATP1A1 and tubulin were used as membrane and cytoplasmic markers, respectively. E. Binding of endogenous ErbB2 to DEPTOR. BT474 whole cell lysates were harvested for IP with anti-ErbB2 Ab, along with normal IgG control, followed by IB with the indicated Abs. WCE: whole-cell extract.

Figure S2. The PDZ domain of DEPTOR binds to ErbB2 and facilitates the membrane localization of DEPTOR

A. DEPTOR binds to ErbB2 via its PDZ domain. HEK293 cells were transfected with the indicated plasmids, followed by IP with FLAG beads and then IB with the indicated Abs. B-C. The PDZ domain facilitates DEPTOR anchorage to cell membrane. HEK293 cells were transfected with the indicated plasmids for 48 hrs, and then subjected to IF and IB with the indicated Abs (B) or subcellular fractionation (C). Scale bars represent 20 μm. Cells showing the plasma membrane localization of DEPTOR or its domains were counted, and their numbers are expressed as the percentage of DEPTOR, or its domains, localized on the plasma membrane.

Figure S3. The stabilization of ErbB2 by DEPTOR overexpression and the effect of mTORC1 activity on ErbB2 stability

A. DEPTOR overexpression stabilizes ErbB2. MCF7 cells infected with retrovirus stably expressing ErbB2 were transfected with indicated plasmids for 48 hrs, and T47D cells were infected with indicated retroviruses to express DEPTOR or ErbB2 for 72 hrs, and then subjected to IB with indicated Abs. B. The effect of mTORC1 activity on ErbB2 stability. BT474 cells infected with lentivirus-based shRNA were pre-incubated with rapamycin for 12 hrs and then treated with CHX for indicated time periods, followed by IB with indicated Abs. Densitometry quantification was performed with Image J, and the decay curves are shown (mean ± S.E.M., n = 3, ***$p < 0.001$) (bottom).

Figure S4. DEPTOR knockdown has no or minor effects on cell proliferation, survival and apoptosis in ErbB2-negative MCF7 cells

MCF7 cells were transfected with indicated siRNA (A), or infected with lentivirus-based shRNA as indicated (B-D), followed by ATPlite cell proliferation assay (A), clonogenic survival assay (B), flow cytometry using the Annexin V-FITC apoptosis detection kit (C) and IB with indicated Abs (D). Shown are mean ± SEM from three independent experiments, n = 3. ns, not significant.

Figure S5. Simultaneous DEPTOR and β-TrCP knockdown does not reverse the changes of cell proliferation and apoptosis induced by DEPTOR knockdown

A. A constitutively active ErbB2 mutant, ErbB2-YVMA, significantly activated the downstream signals of ErbB2. HEK293 cells were transfected with indicated plasmids, followed by IB with indicated Abs. B-E. Simultaneous DEPTOR and β-TrCP knockdown does not reverse the changes of cell proliferation and apoptosis induced by DEPTOR knockdown. BT474 cells were transfected with indicated siRNA (B) or infected with lentivirus-based shRNA as indicated (C-E), followed by ATPlite cell proliferation assay (B) and clonogenic survival assay (C), flow cytometry using the Annexin V-FITC apoptosis detection kit (D) and IB with indicated Abs (E). Shown are mean ± SEM from three independent experiments, n = 3; ns, not significant, *$p < 0.05$, ***$p < 0.001$. F. Binding of endogenous DEPTOR to β-TrCP. BT474 whole cell lysates were harvested for IP with anti-DEPTOR Ab, along with normal IgG control, followed by IB with the indicated Abs. WCE: whole-cell extract.

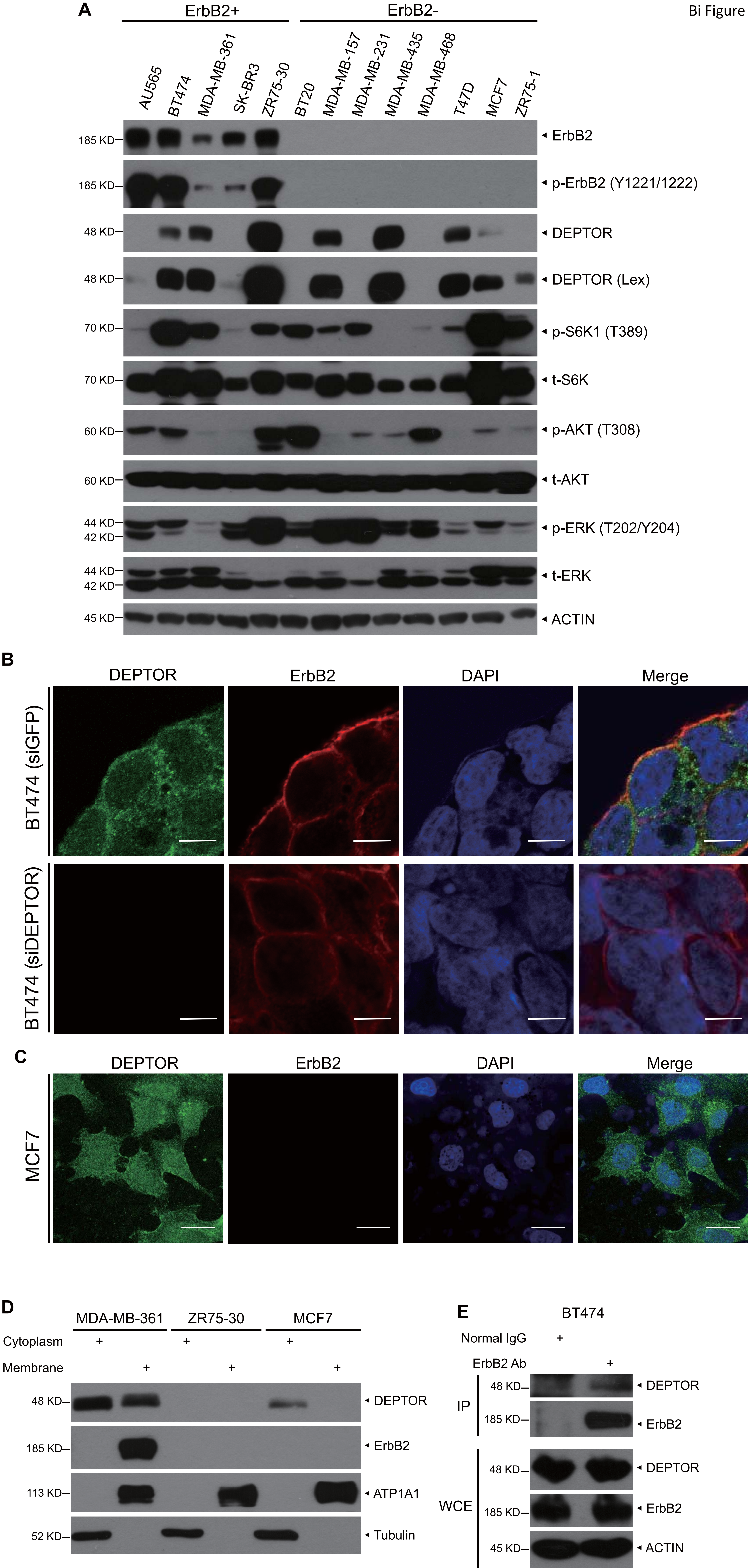
A
ErbB2+
ErbB2-
AU565
BT474
MDA-MB-361
SK-BR3
ZR75-30
BT20
MDA-MB-157
MDA-MB-231
MDA-MB-435
MDA-MB-468
T47D
MCF7
ZR75-1
185 KD
ErbB2
185 KD
p-ErbB2 (Y1221/1222)
48 KD
DEPTOR
48 KD
DEPTOR (Lex)
70 KD
p-S6K1 (T389)
70 KD
t-S6K
60 KD
p-AKT (T308)
60 KD
t-AKT
44 KD
42 KD
p-ERK (T202/Y204)
44 KD
42 KD
t-ERK
45 KD
ACTIN
B
DEPTOR
ErbB2
DAPI
Merge
BT474 (siGFP)
BT474 (siDEPTOR)
C
DEPTOR
ErbB2
DAPI
Merge
MCF7
D
MDA-MB-361
ZR75-30
MCF7
Cytoplasm
Membrane
48 KD
DEPTOR
185 KD
ErbB2
113 KD
ATP1A1
52 KD
Tubulin
E
BT474
Normal IgG
ErbB2 Ab
IP
48 KD
DEPTOR
185 KD
ErbB2
WCE
48 KD
DEPTOR
185 KD
ErbB2
45 KD
ACTIN

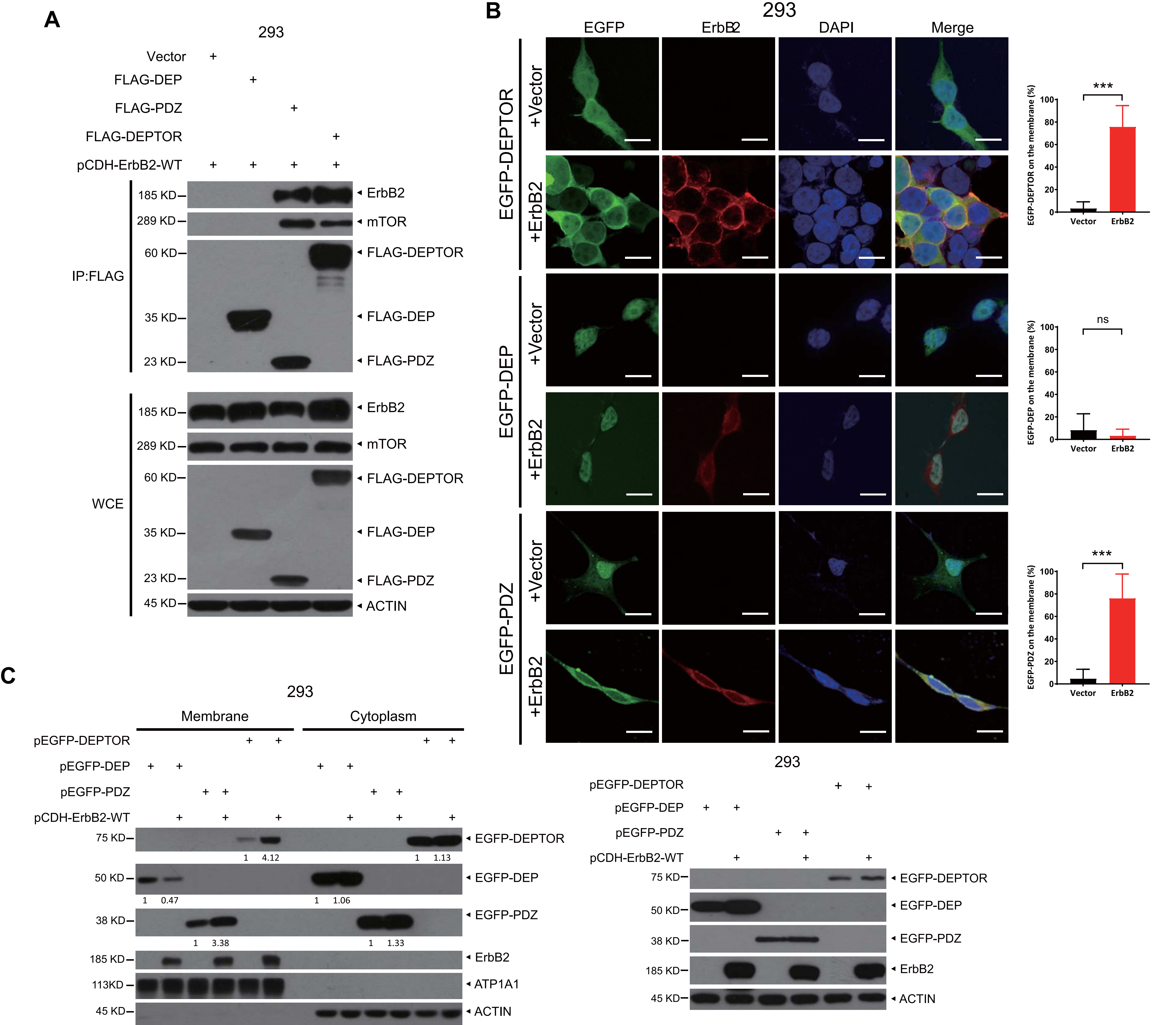
A
293
Vector
FLAG-DEP
FLAG-PDZ
FLAG-DEPTOR
pCDH-ErbB2-WT
IP:FLAG
185 KD
289 KD
60 KD
35 KD
23 KD
ErbB2
mTOR
FLAG-DEPTOR
FLAG-DEP
FLAG-PDZ
WCE
45 KD
ACTIN
B
293
EGFP
ErbB2
DAPI
Merge
EGFP-DEPTOR
EGFP-DEP
EGFP-PDZ
+Vector
+ErbB2
EGFP-DEPTOR on the membrane (%)
EGFP-DEP on the membrane (%)
EGFP-PDZ on the membrane (%)
***
ns
Vector
ErbB2
C
293
Membrane
Cytoplasm
pEGFP-DEPTOR
pEGFP-DEP
pEGFP-PDZ
pCDH-ErbB2-WT
75 KD
50 KD
38 KD
185 KD
113KD
45 KD
EGFP-DEPTOR
EGFP-DEP
EGFP-PDZ
ErbB2
ATP1A1
ACTIN
1 4.12
1 1.13
1 0.47
1 1.06
1 3.38
1 1.33

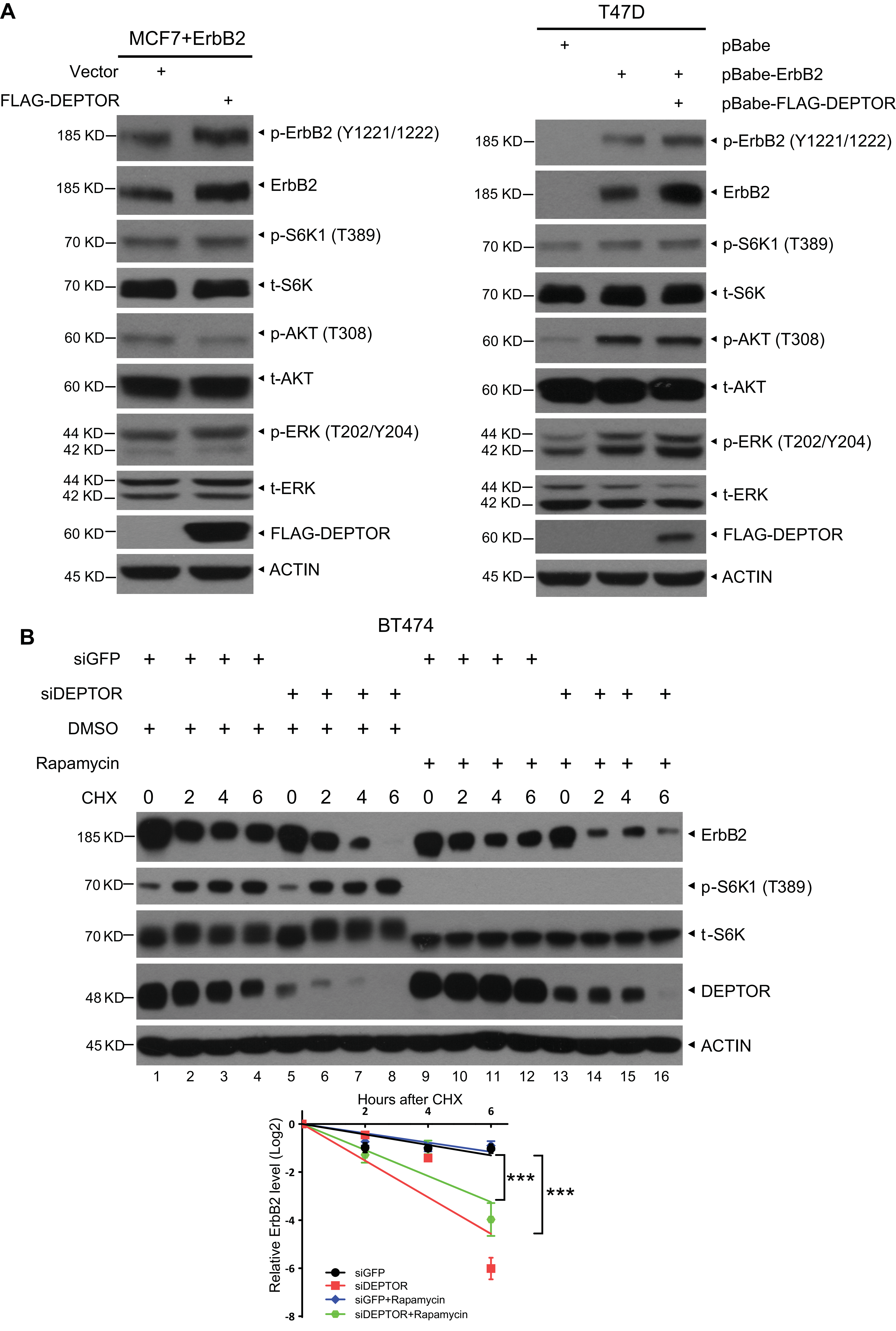
A
MCF7+ErbB2
Vector
FLAG-DEPTOR
185 KD
p-ErbB2 (Y1221/1222)
ErbB2
70 KD
p-S6K1 (T389)
t-S6K
60 KD
p-AKT (T308)
t-AKT
44 KD
42 KD
p-ERK (T202/Y204)
t-ERK
FLAG-DEPTOR
45 KD
ACTIN
T47D
pBabe
pBabe-ErbB2
pBabe-FLAG-DEPTOR
B
BT474
siGFP
siDEPTOR
DMSO
Rapamycin
CHX
0 2 4 6 0 2 4 6 0 2 4 6 0 2 4 6
48 KD
DEPTOR
1 2 3 4 5 6 7 8 9 10 11 12 13 14 15 16
Hours after CHX
Relative ErbB2 level (Log2)
***
***
siGFP
siDEPTOR
siGFP+Rapamycin
siDEPTOR+Rapamycin

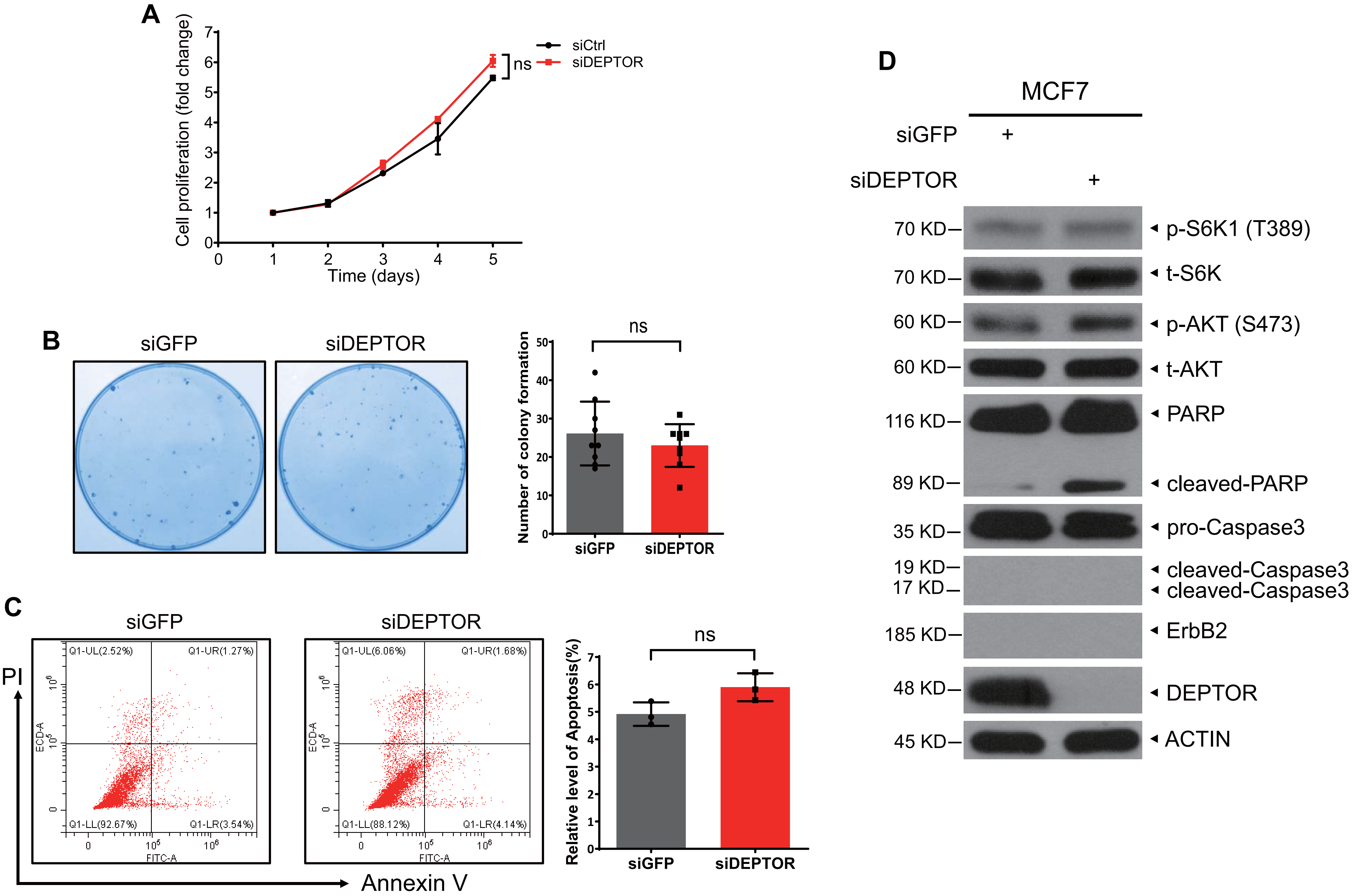
A
Cell proliferation (fold change)
Time (days)
siCtrl
siDEPTOR
ns
B
siGFP
siDEPTOR
Number of colony formation
ns
C
siGFP
siDEPTOR
Q1-UL(2.52%)
Q1-UR(1.27%)
Q1-LL(92.67%)
Q1-LR(3.54%)
Q1-UL(6.06%)
Q1-UR(1.68%)
Q1-LL(88.12%)
Q1-LR(4.14%)
ECD-A
FITC-A
PI
Annexin V
Relative level of Apoptosis(%)
ns
D
MCF7
siGFP
siDEPTOR
70 KD
p-S6K1 (T389)
70 KD
t-S6K
60 KD
p-AKT (S473)
60 KD
t-AKT
116 KD
PARP
89 KD
cleaved-PARP
35 KD
pro-Caspase3
19 KD
cleaved-Caspase3
17 KD
cleaved-Caspase3
185 KD
ErbB2
48 KD
DEPTOR
45 KD
ACTIN

Bi Figure S5

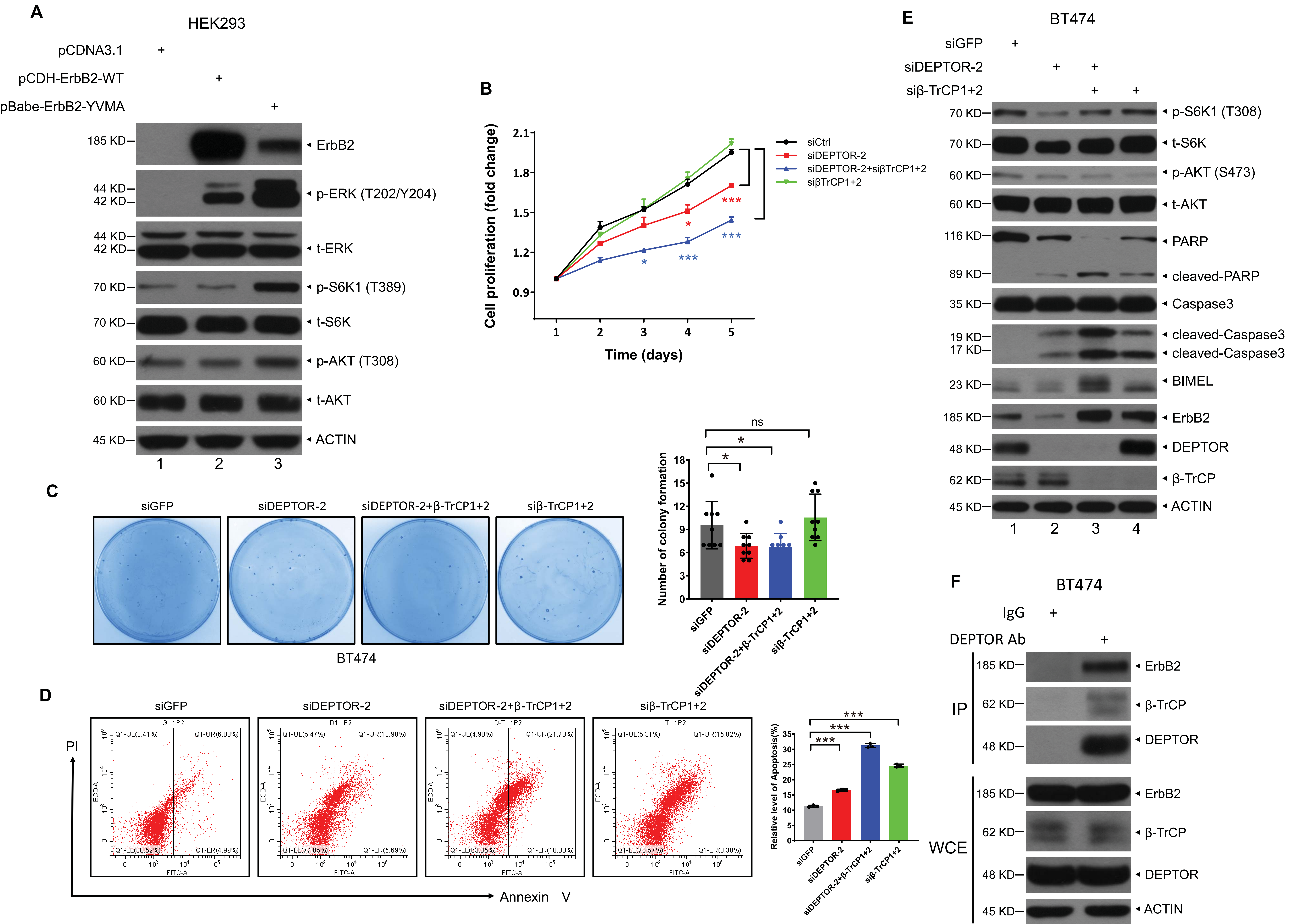

A
HEK293
pCDNA3.1
pCDH-ErbB2-WT
pBabe-ErbB2-YVMA
185 KD
ErbB2
44 KD
42 KD
p-ERK (T202/Y204)
t-ERK
70 KD
p-S6K1 (T389)
t-S6K
60 KD
p-AKT (T308)
t-AKT
45 KD
ACTIN
1 2 3
B
Cell proliferation (fold change)
Time (days)
siCtrl
siDEPTOR-2
siDEPTOR-2+siβTrCP1+2
siβTrCP1+2
C
siGFP
siDEPTOR-2
siDEPTOR-2+β-TrCP1+2
siβ-TrCP1+2
BT474
Number of colony formation
ns
siGFP
siDEPTOR-2
siDEPTOR-2+β-TrCP1+2
siβ-TrCP1+2
D
siGFP
siDEPTOR-2
siDEPTOR-2+β-TrCP1+2
siβ-TrCP1+2
PI
Annexin V
Q1-UL(0.41%)
Q1-UR(6.08%)
Q1-LL(88.52%)
Q1-LR(4.99%)
Q1-UL(5.47%)
Q1-UR(10.98%)
Q1-LL(77.85%)
Q1-LR(5.69%)
Q1-UL(4.90%)
Q1-UR(21.73%)
Q1-LL(63.05%)
Q1-LR(10.33%)
Q1-UL(5.31%)
Q1-UR(15.82%)
Q1-LL(70.57%)
Q1-LR(8.30%)
FITC-A
ECD-A
Relative level of Apoptosis(%)
E
BT474
siGFP
siDEPTOR-2
siβ-TrCP1+2
p-S6K1 (T308)
t-S6K
p-AKT (S473)
t-AKT
116 KD
PARP
89 KD
cleaved-PARP
35 KD
Caspase3
19 KD
17 KD
cleaved-Caspase3
cleaved-Caspase3
23 KD
BIMEL
185 KD
ErbB2
48 KD
DEPTOR
62 KD
β-TrCP
45 KD
ACTIN
1 2 3 4
F
BT474
IgG
DEPTOR Ab
IP
WCE
ErbB2
β-TrCP
DEPTOR
ACTIN

Original IBs

Figure 2C

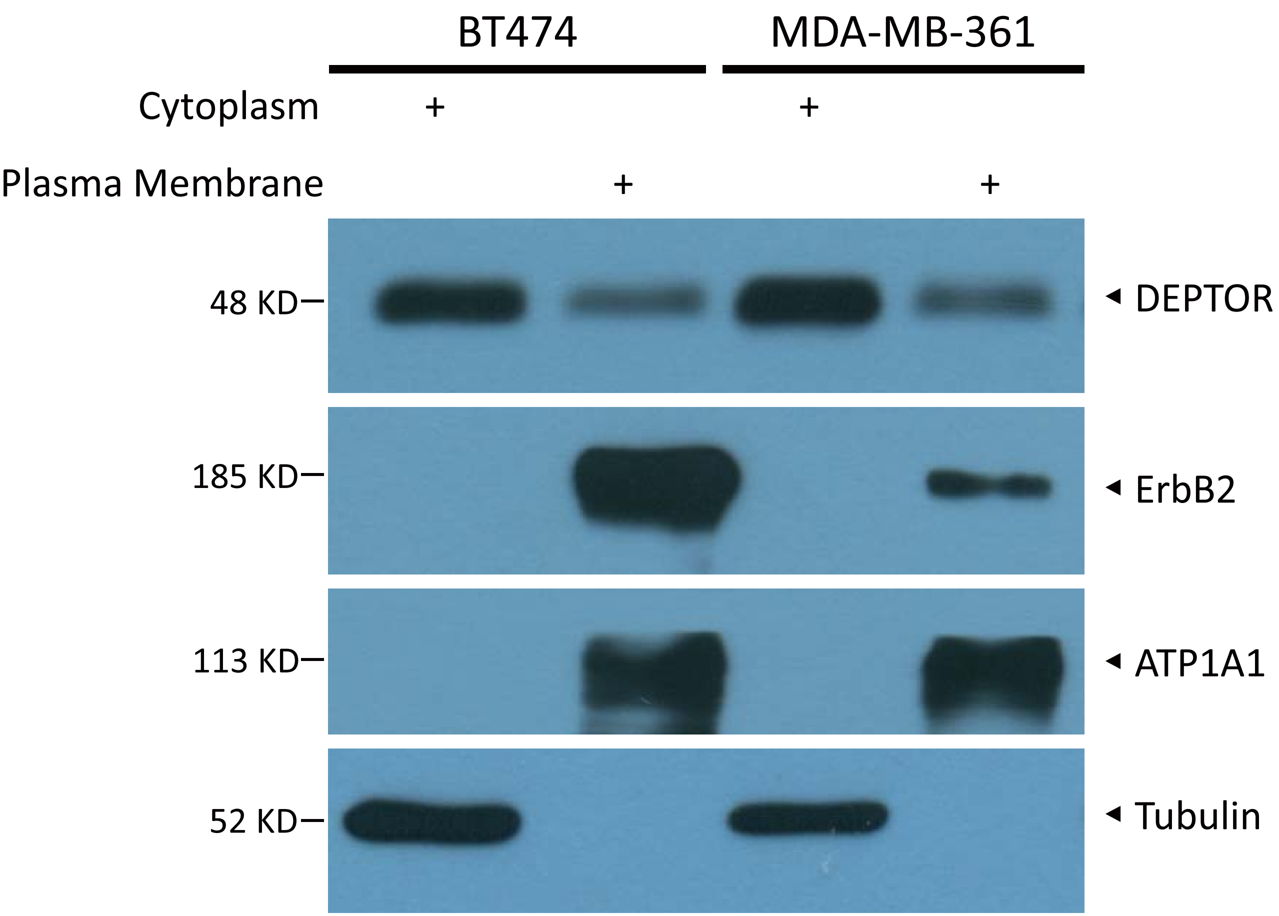

Figure 2D

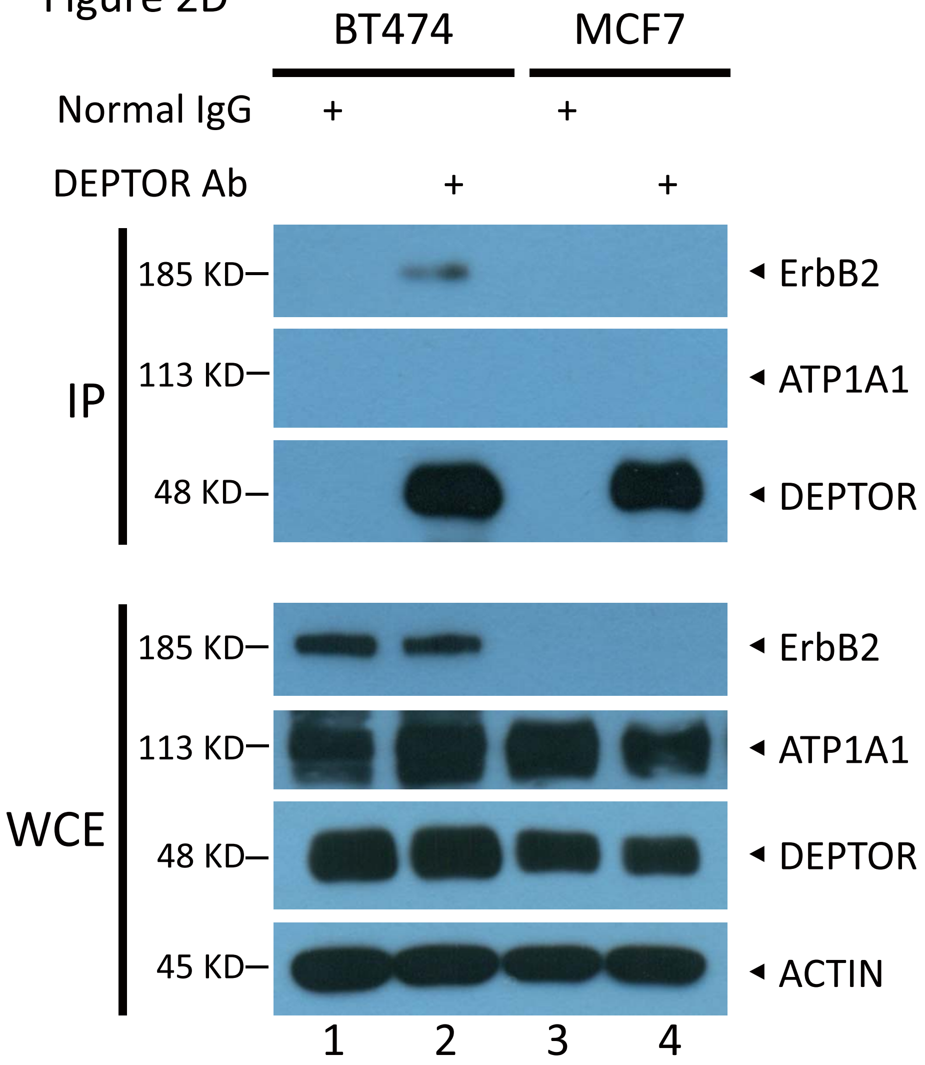

Figure 2E

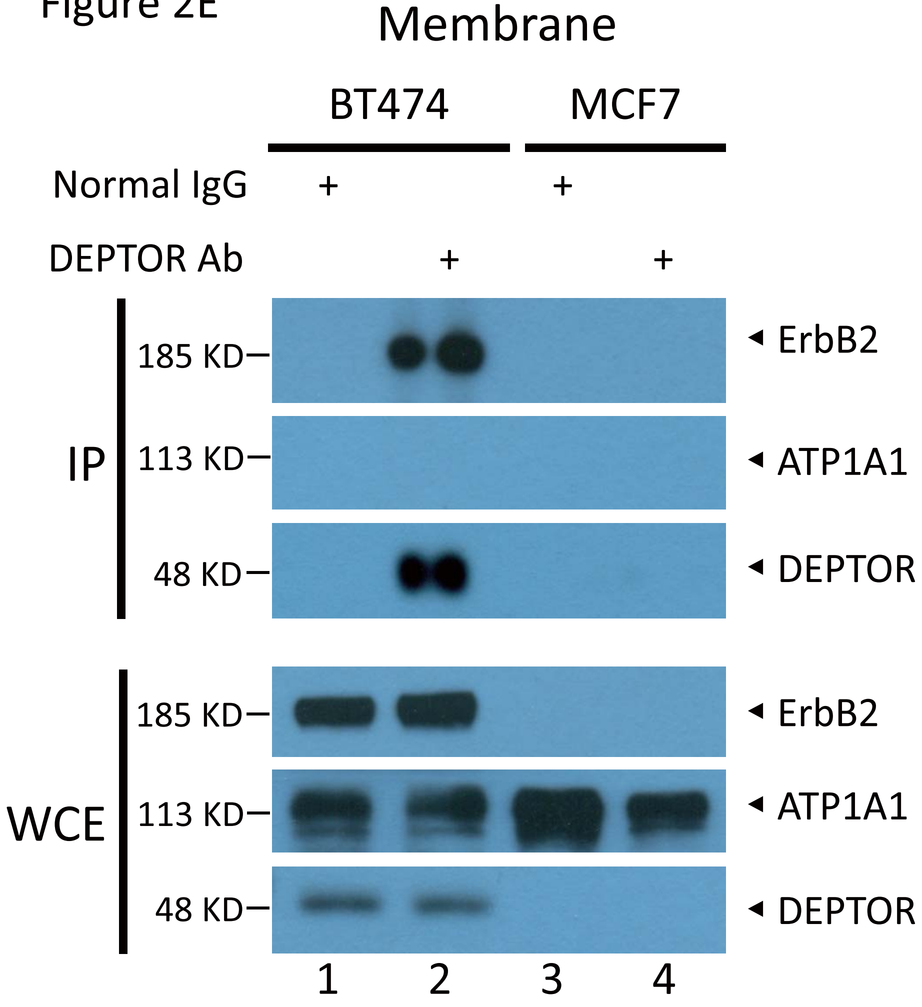

Original IBs

Figure 3A

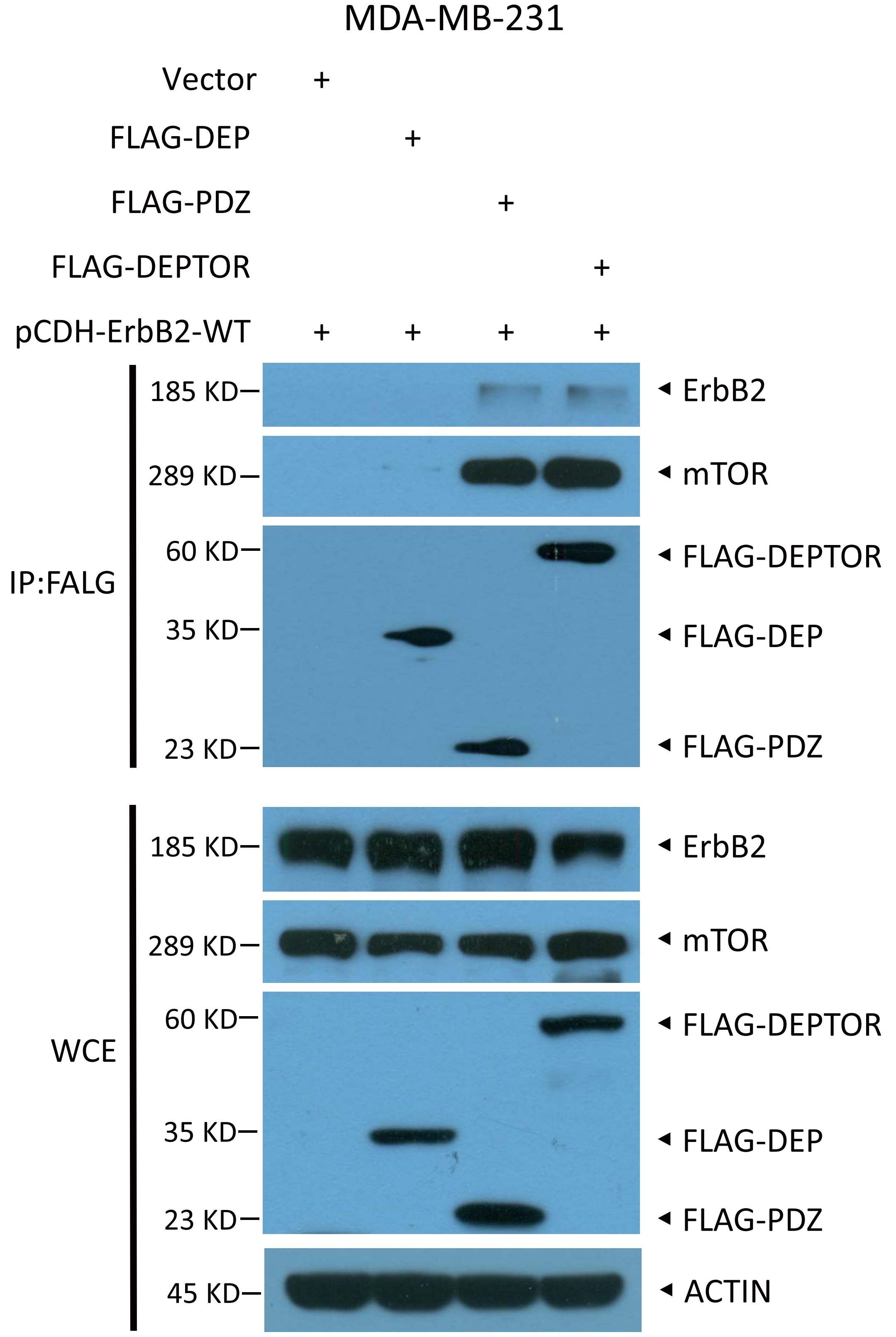

Figure 3B

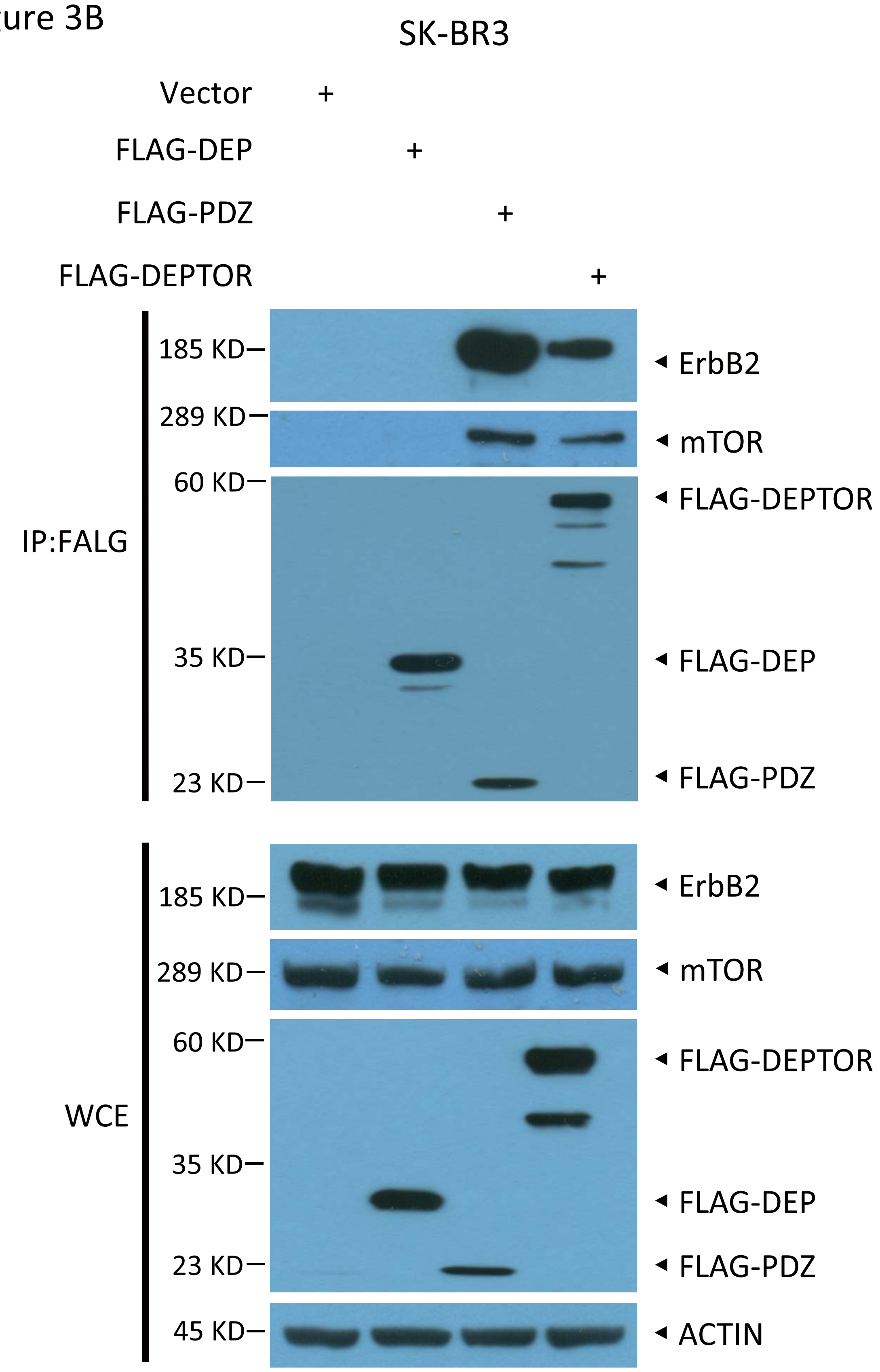

Figure 3C

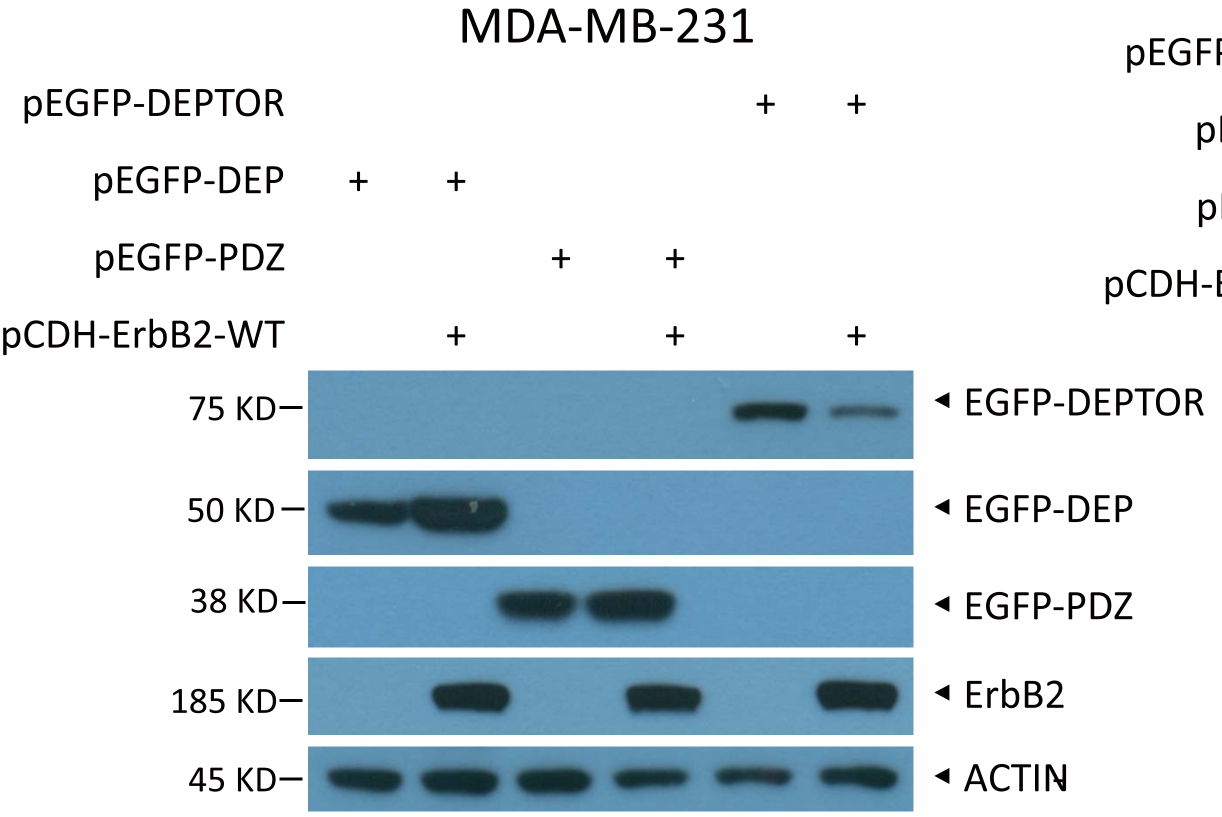

Figure 3D

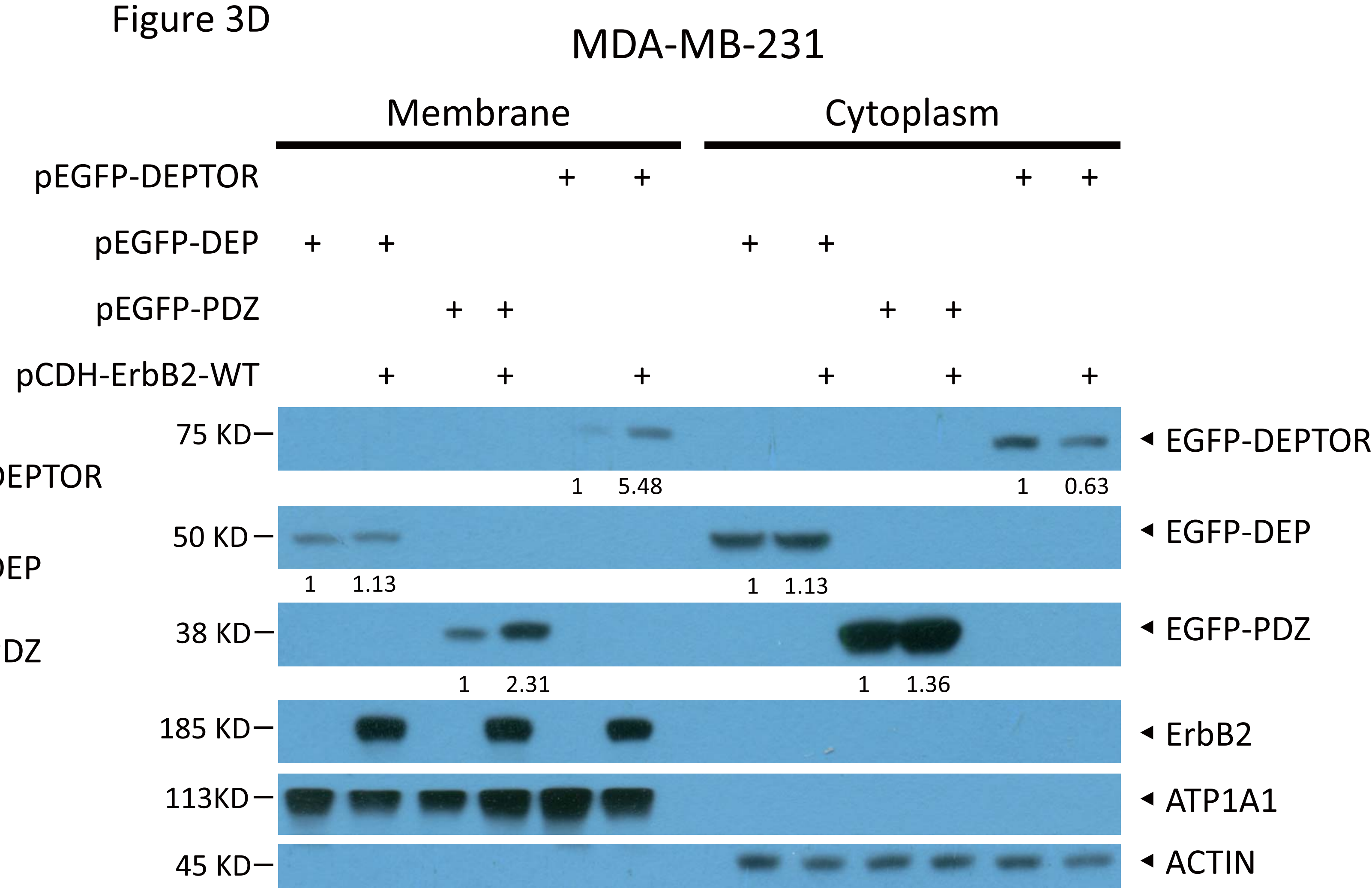

Original IBs

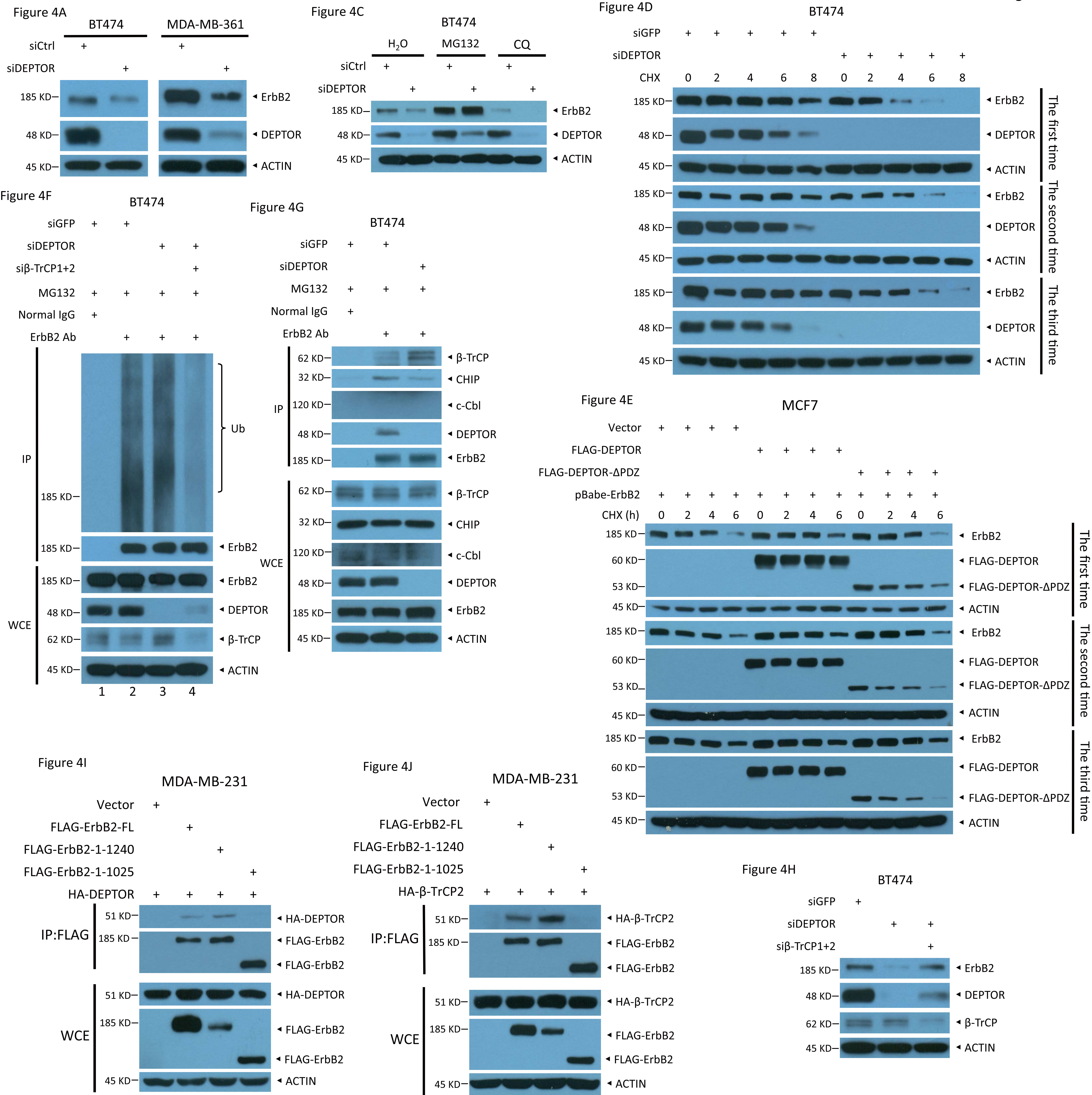

Original IBs

Figure 5C

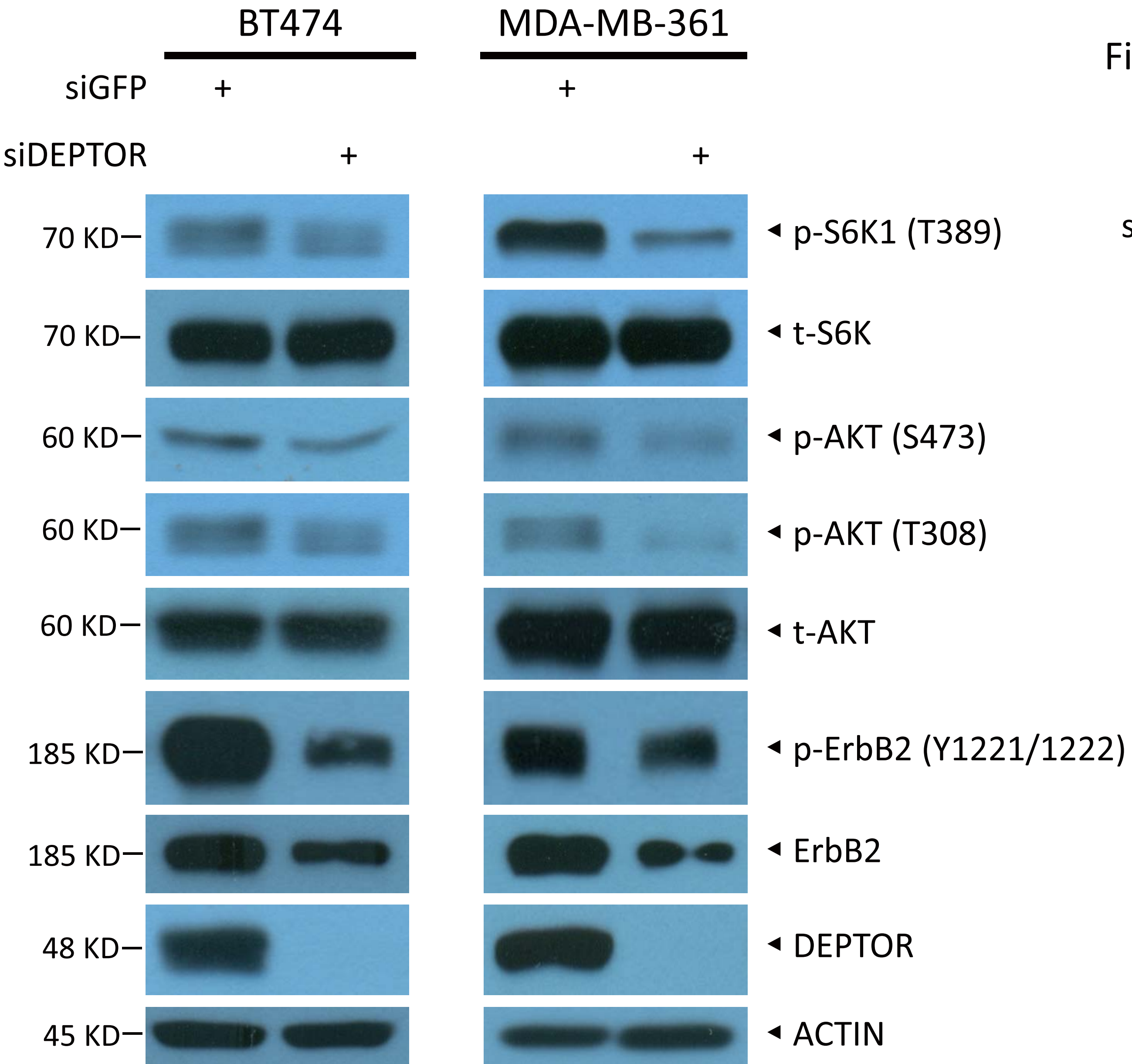

Figure 5E

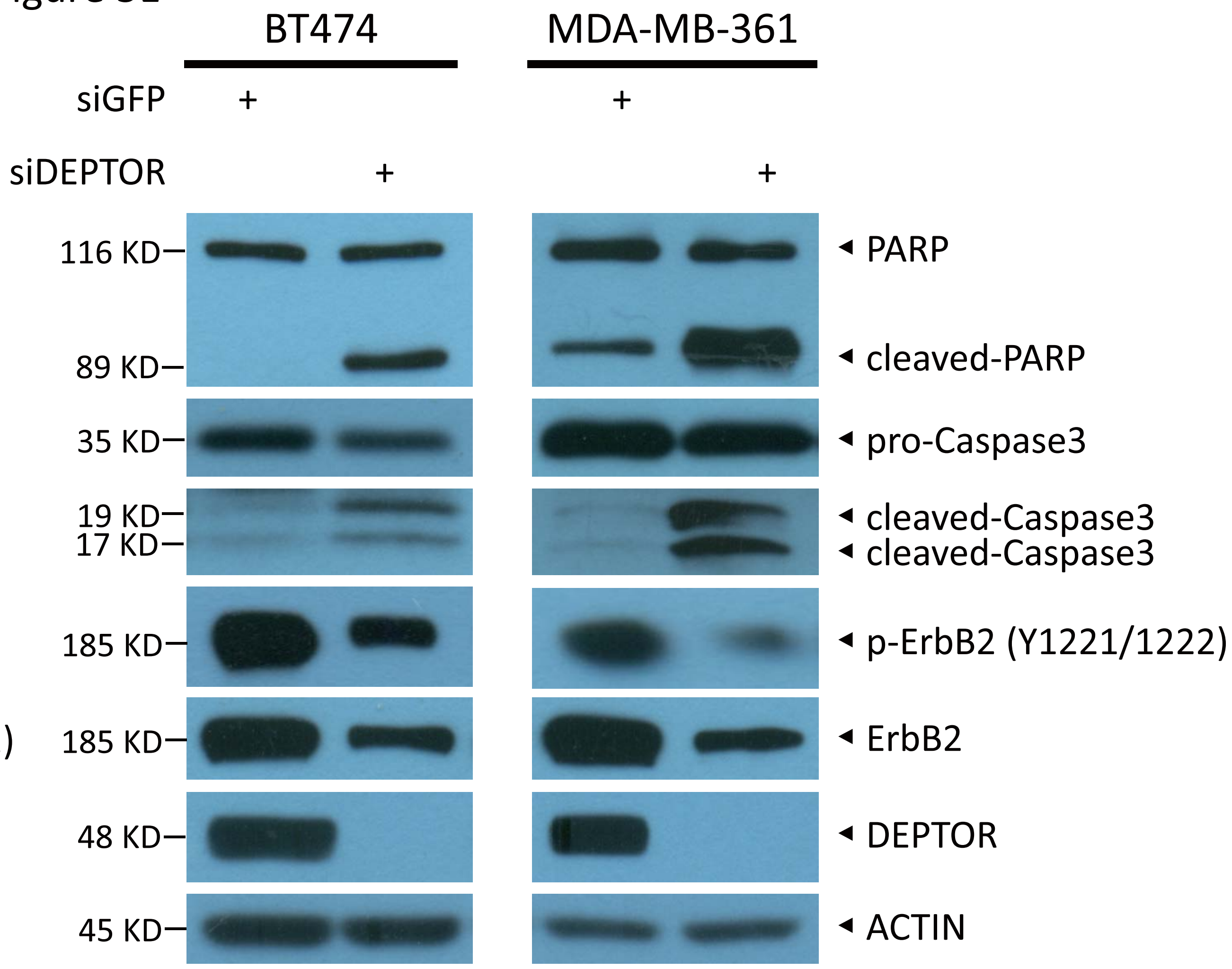

Original IBs

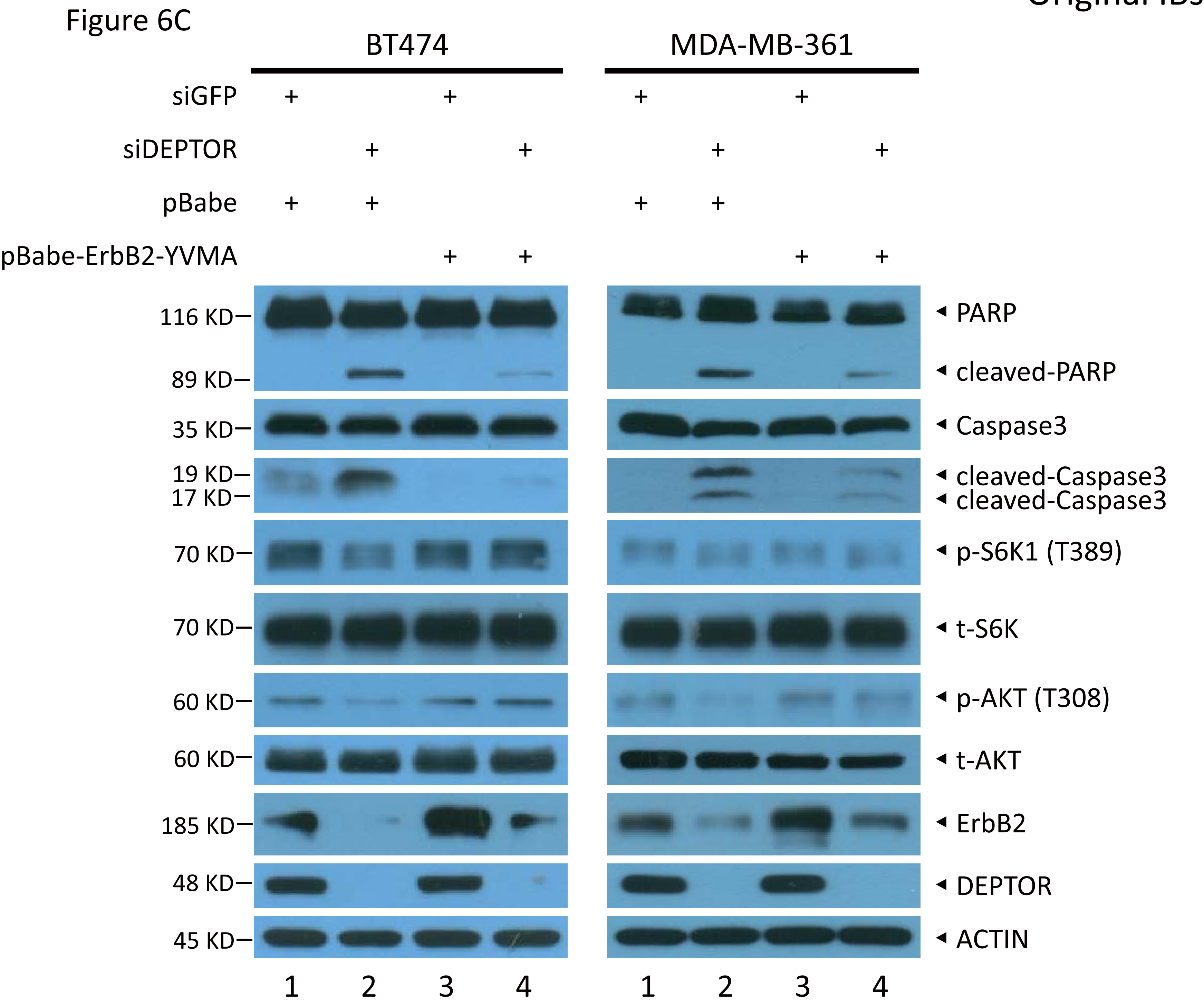

Original IBs

Figure S1A

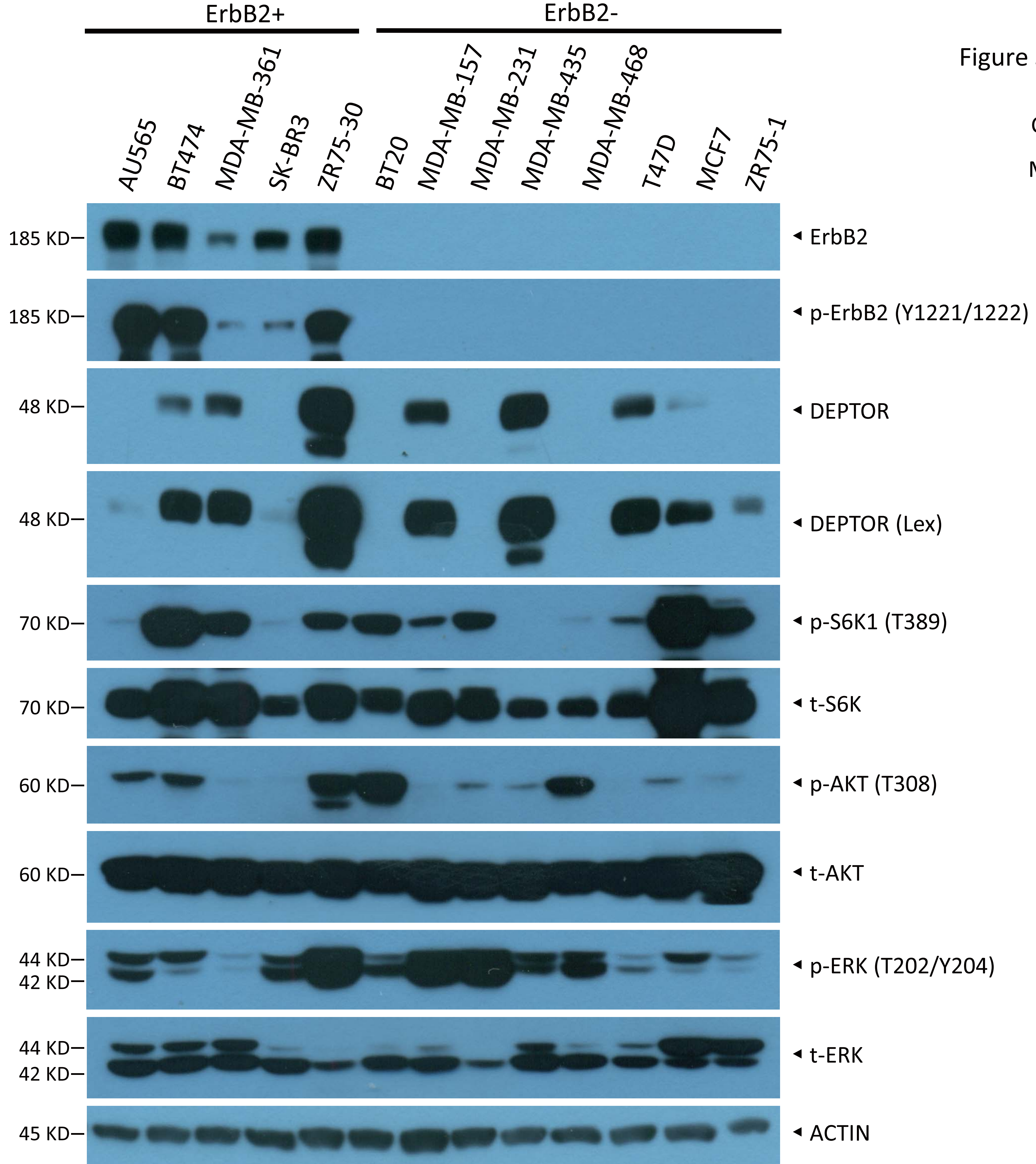

Figure S1C

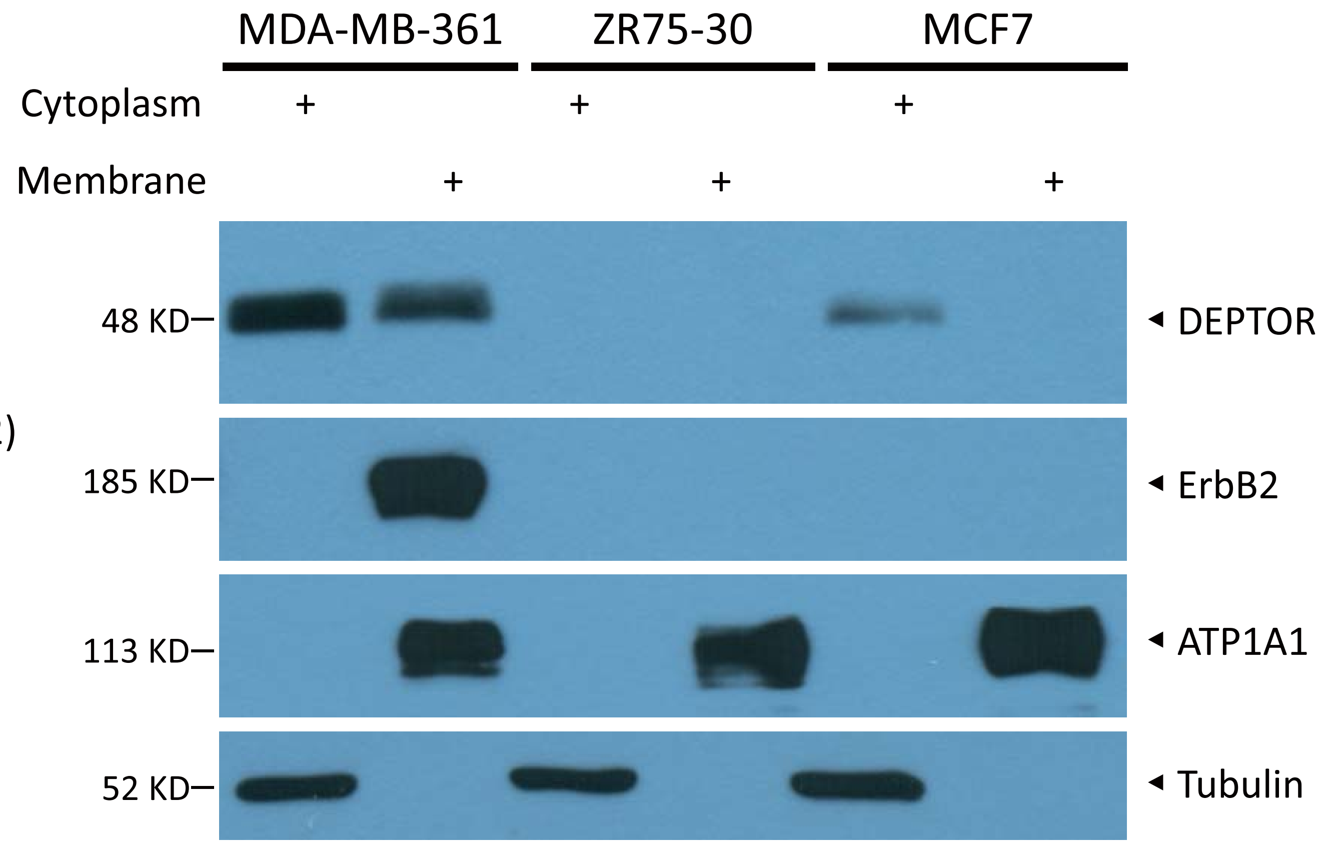

Figure S1E

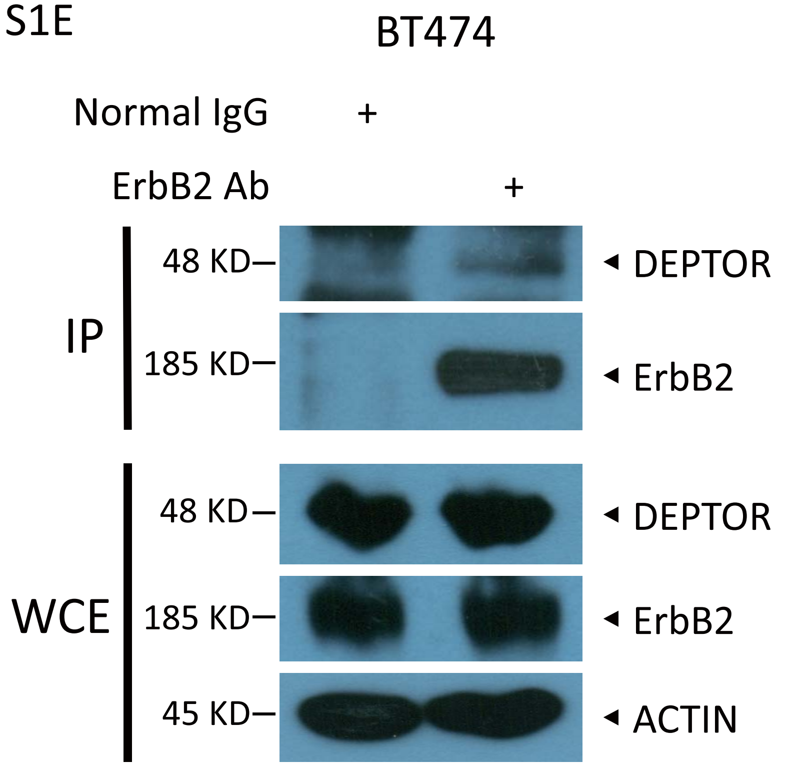

Original IBs

Figure S2A

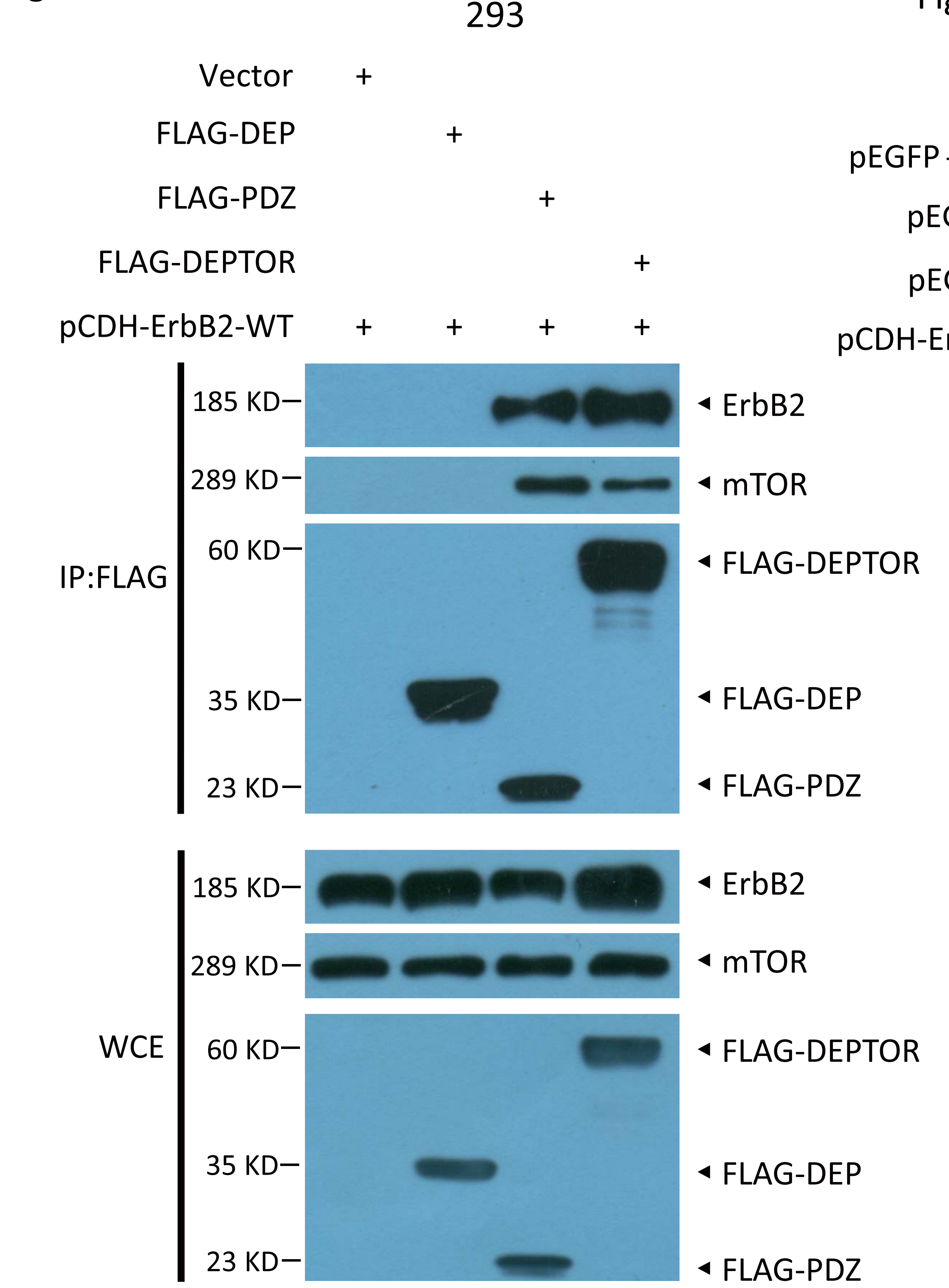

Figure S2B

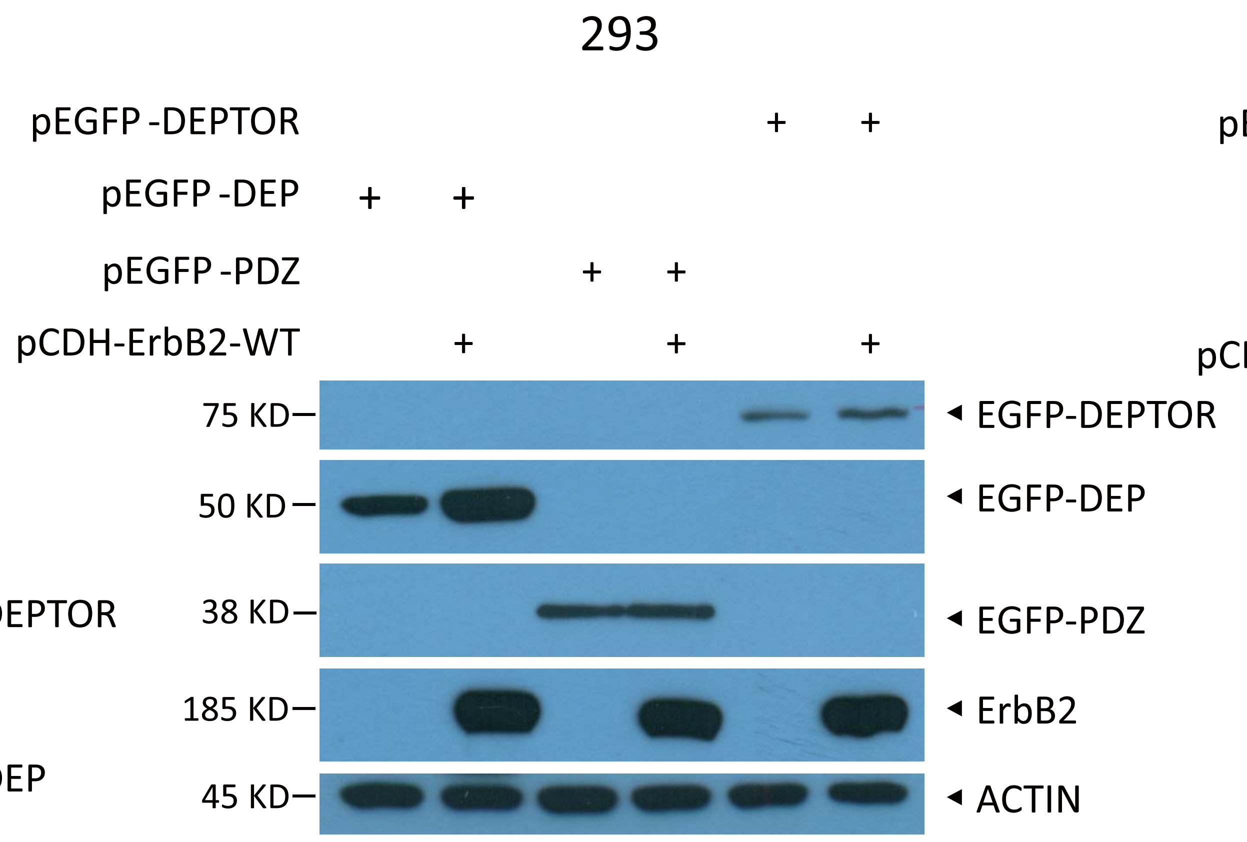

Figure S2C

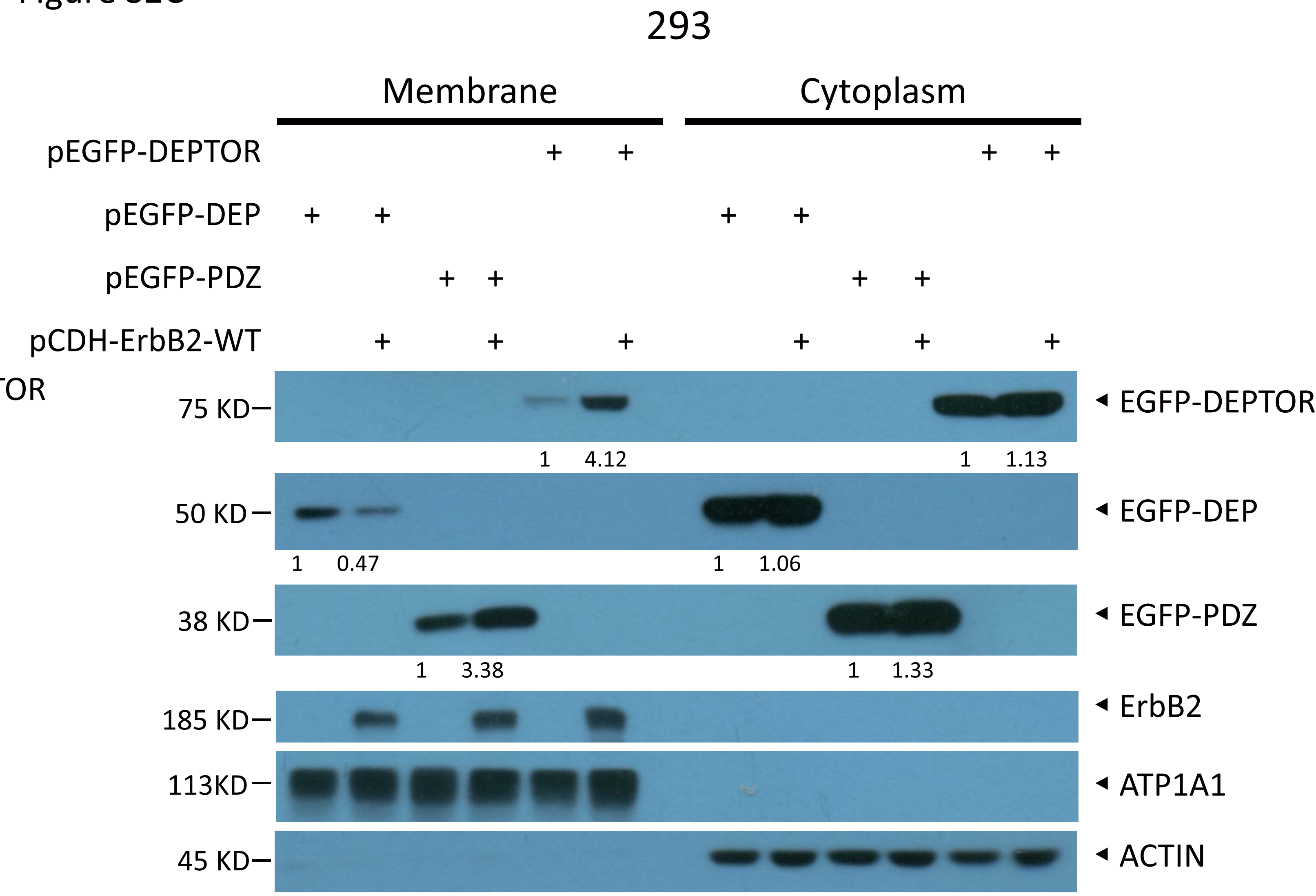

Original IBs

Figure S3A

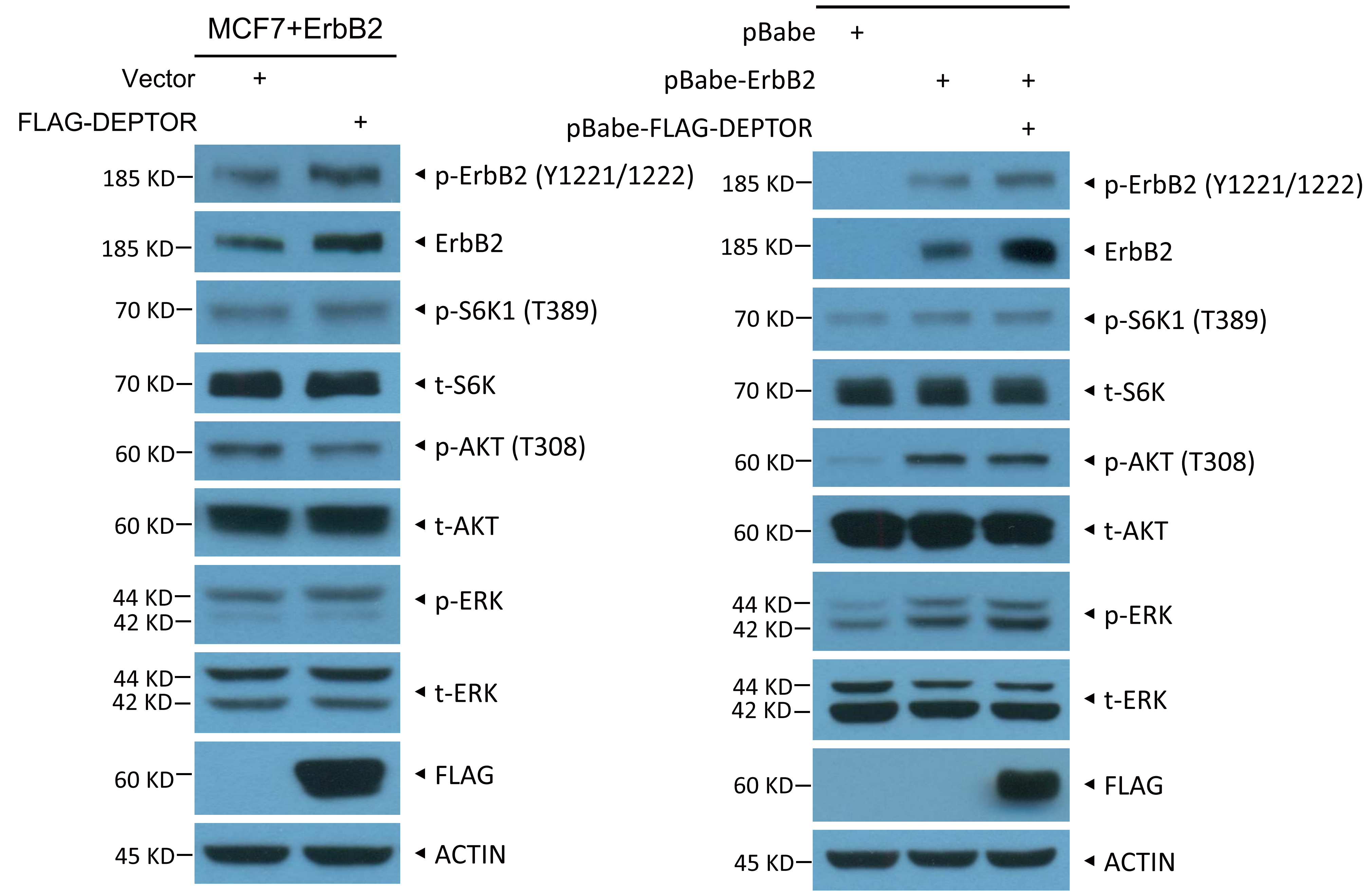

Figure S3B

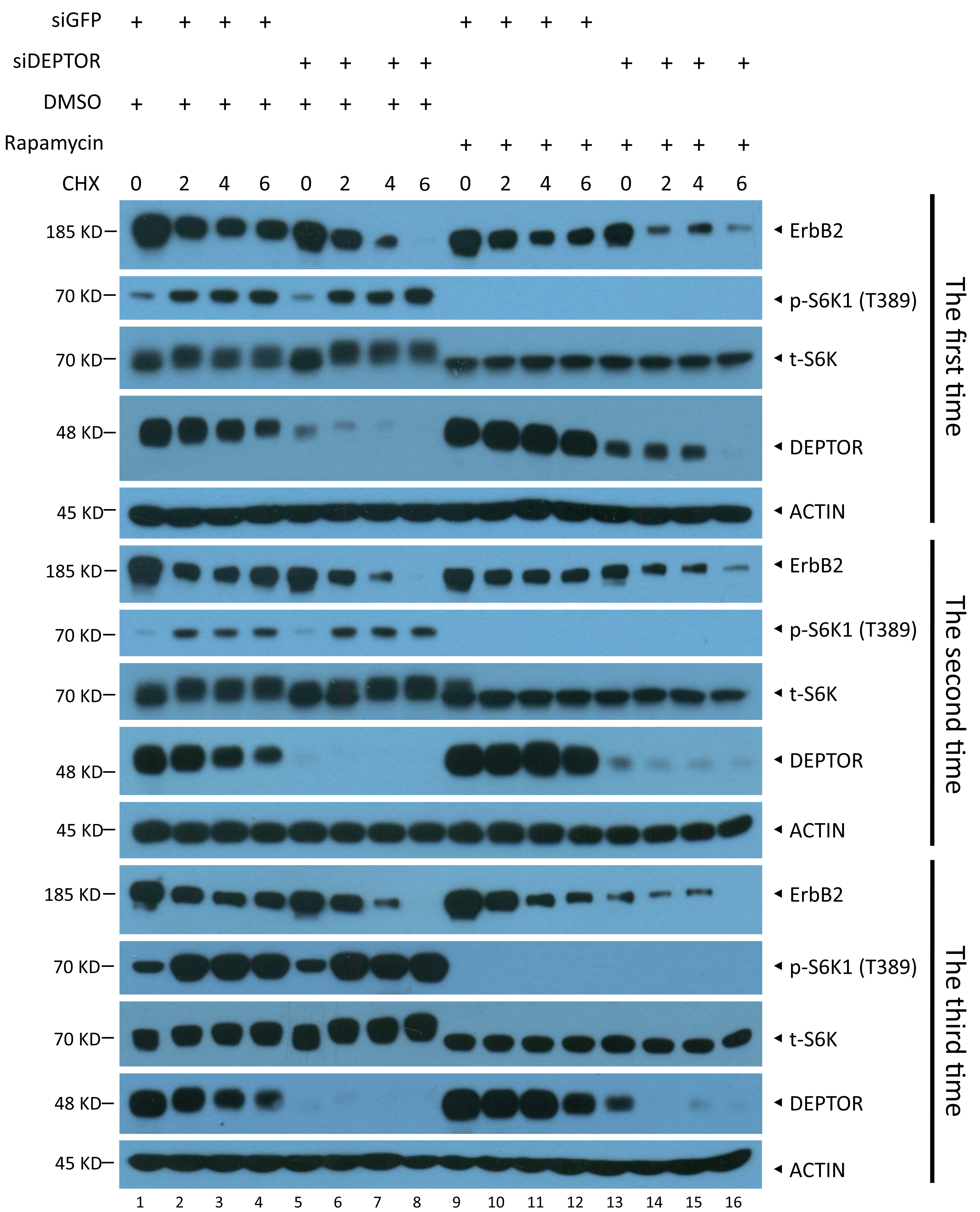

Original IBs

Figure S4D

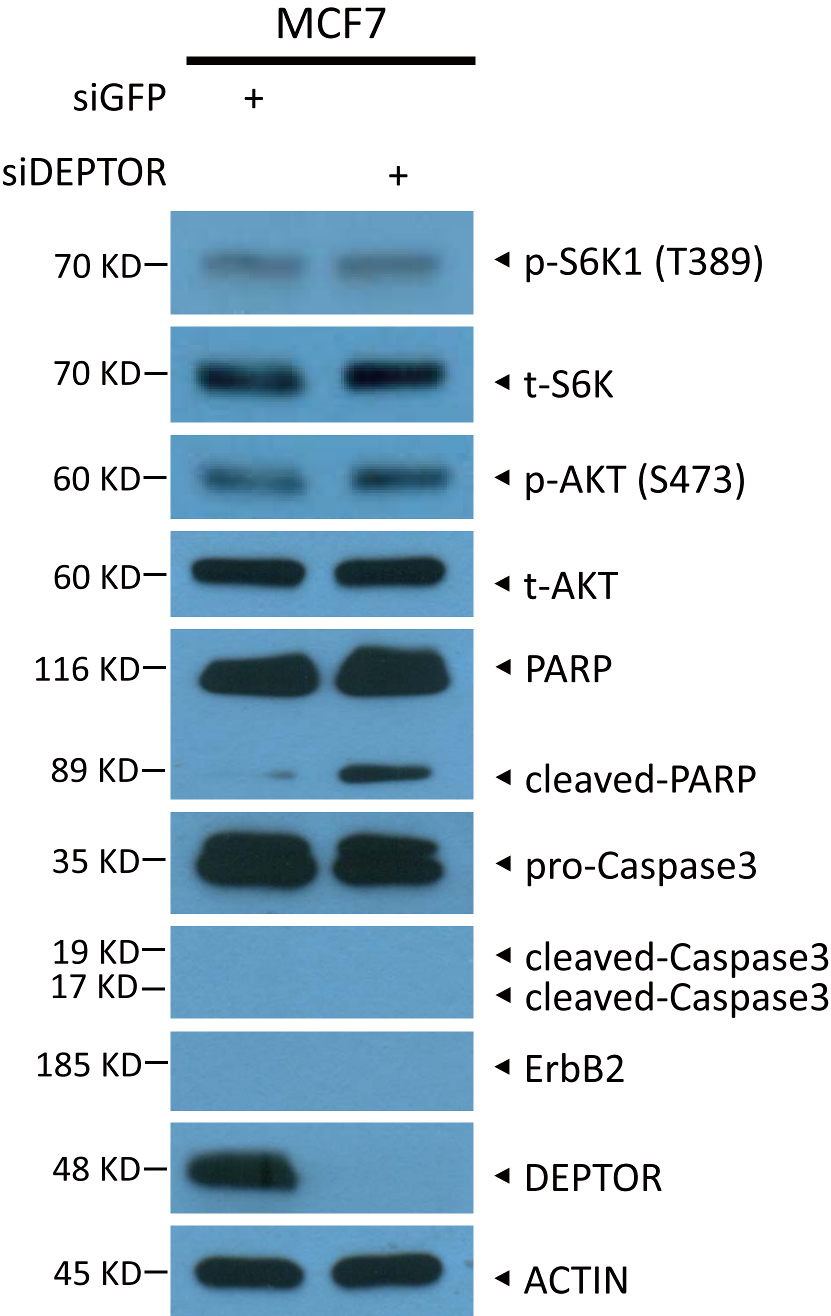

Original IBs

Figure S5A

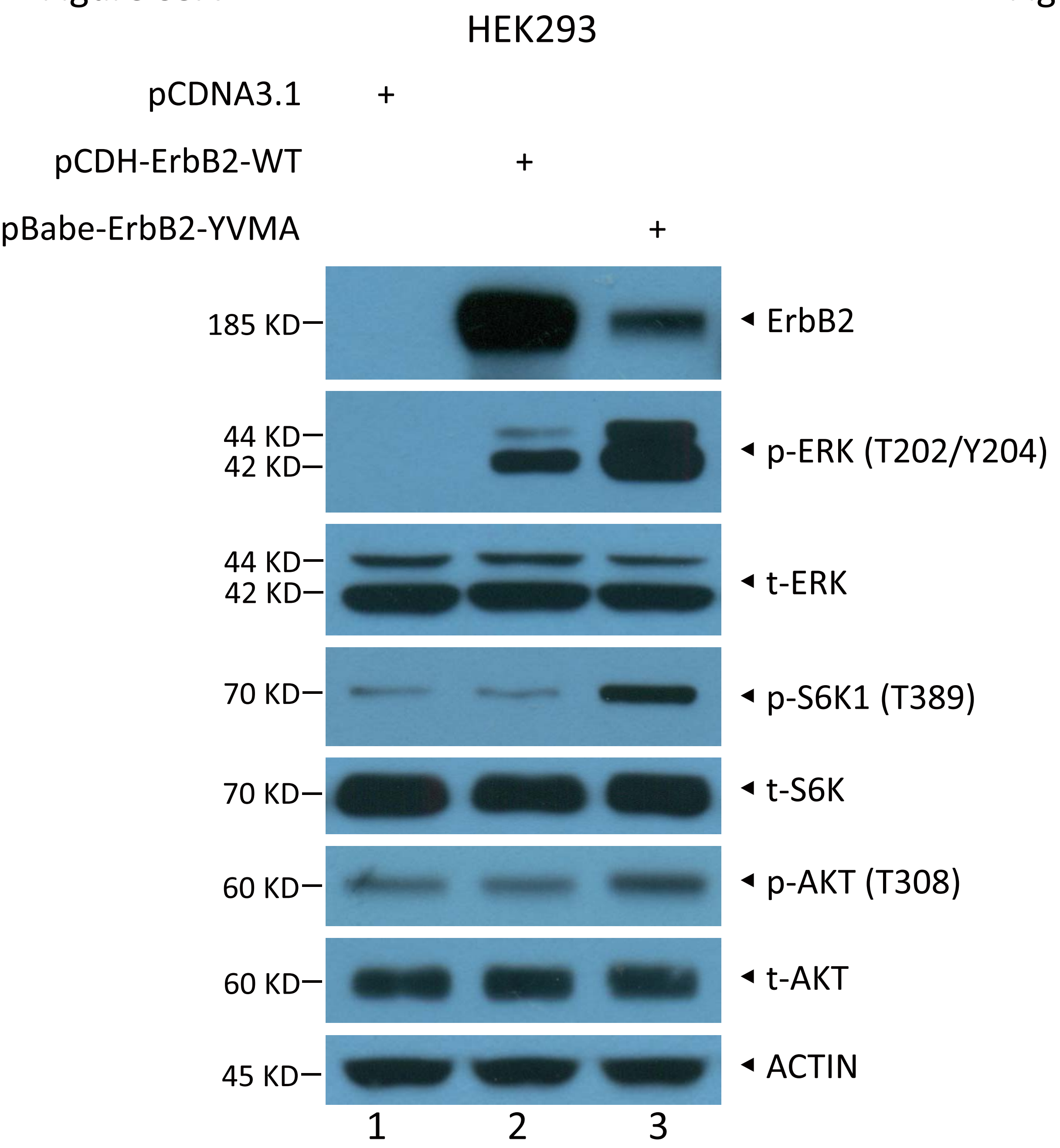

Figure S5E

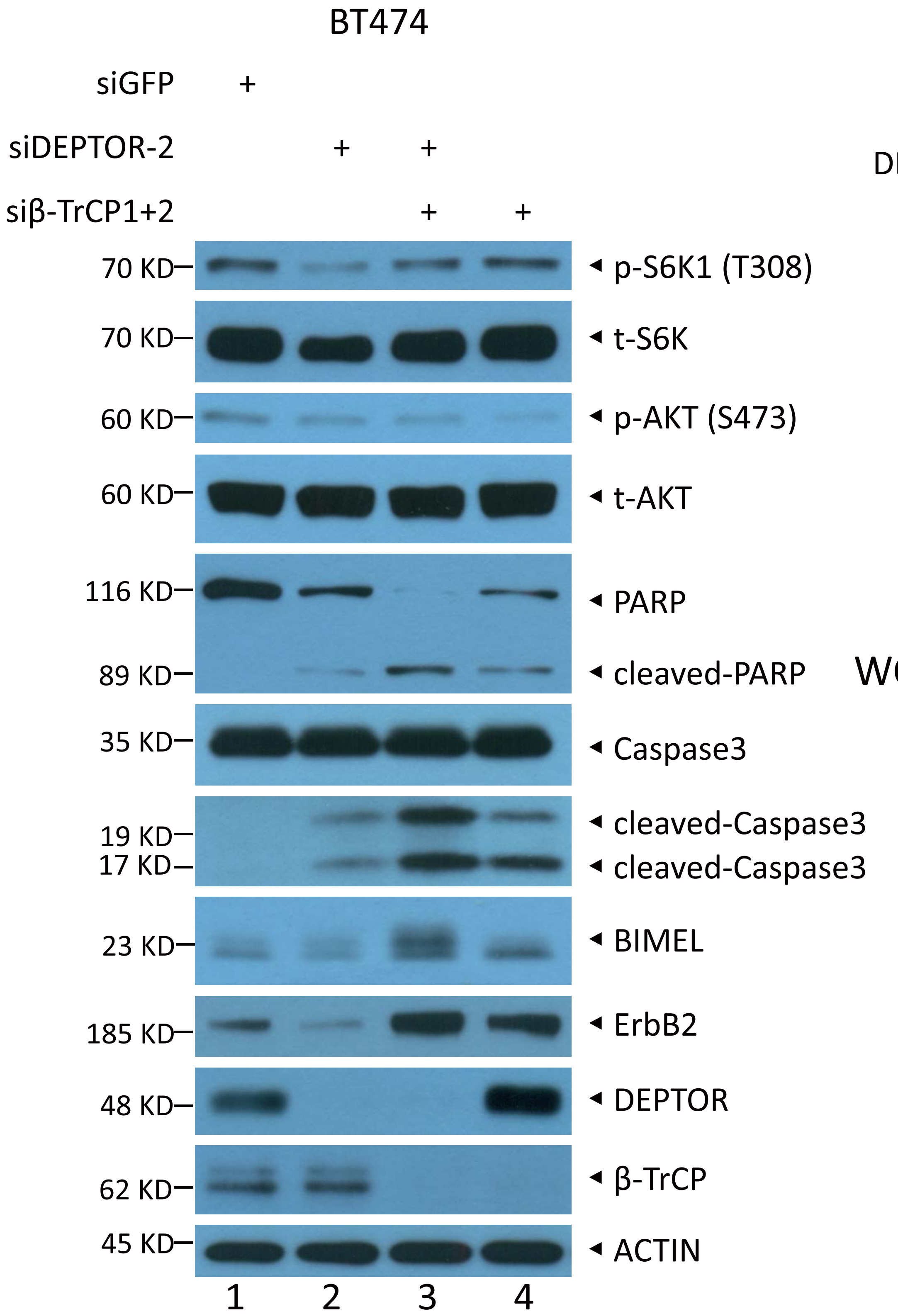

Figure S5F

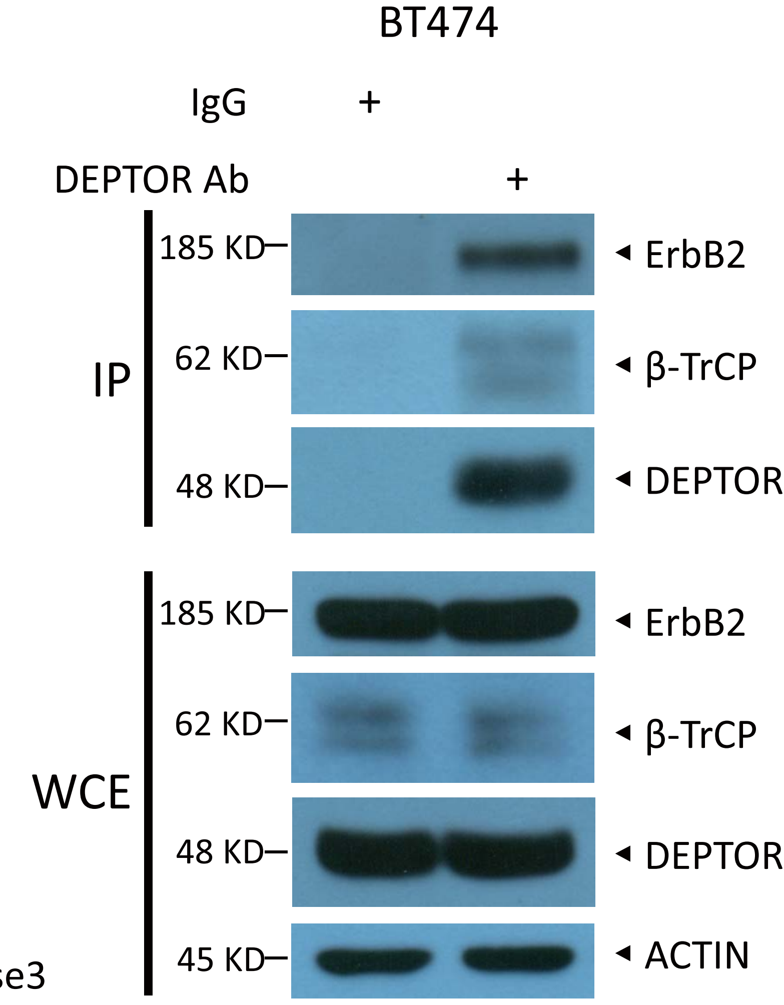

Supplement: Supplementary file 1 — Supplementary figures. [file thnov11p6355s1.pdf]
